# MetaDecoder: a novel method for clustering metagenomic contigs

**DOI:** 10.1186/s40168-022-01237-8

**Published:** 2022-03-10

**Authors:** Cong-Cong Liu, Shan-Shan Dong, Jia-Bin Chen, Chen Wang, Pan Ning, Yan Guo, Tie-Lin Yang

**Affiliations:** 1 Key Laboratory of Biomedical Information Engineering of Ministry of Education, Biomedical Informatics & Genomics Center, School of Life Science and Technology, Xi’an Jiaotong University, Xi’an, Shaanxi 710049 P. R. China; 2 Key Laboratory of Biomedical Information Engineering of Ministry of Education, School of Life Science and Technology, Xi’an Jiaotong University, Xi’an, Shaanxi 710049 P. R. China; 3 National and Local Joint Engineering Research Center of Biodiagnosis and Biotherapy, The Second Affiliated Hospital, Xi’an Jiaotong University, Xi’an, Shaanxi 710004 P. R. China

**Keywords:** MetaDecoder, Clustering algorithm, Metagenome, DPGMM, GMM

## Abstract

**Background:**

Clustering the metagenomic contigs into potential genomes is a key step to investigate the functional roles of microbial populations. Existing algorithms have achieved considerable success with simulated or real sequencing datasets. However, accurately classifying contigs from complex metagenomes is still a challenge.

**Results:**

We introduced a novel clustering algorithm, MetaDecoder, which can classify metagenomic contigs based on the frequencies of *k*-mers and coverages. MetaDecoder was built as a two-layer model with the first layer being a GPU-based modified Dirichlet process Gaussian mixture model (DPGMM), which controls the weight of each DPGMM cluster to avoid over-segmentation by dynamically dissolving contigs in small clusters and reassigning them to the remaining clusters. The second layer comprises a semi-supervised *k*-mer frequency probabilistic model and a modified Gaussian mixture model for modeling the coverage based on single copy marker genes. Benchmarks on simulated and real-world datasets demonstrated that MetaDecoder can be served as a promising approach for effectively clustering metagenomic contigs.

**Conclusions:**

In conclusion, we developed the GPU-based MetaDecoder for effectively clustering metagenomic contigs and reconstructing microbial communities from microbial data. Applying MetaDecoder on both simulated and real-world datasets demonstrated that it could generate more complete clusters with lower contamination. Using MetaDecoder, we identified novel high-quality genomes and expanded the existing catalog of bacterial genomes.

Video Abstract

**Supplementary Information:**

The online version contains supplementary material available at 10.1186/s40168-022-01237-8.

## Background

Shotgun sequencing has been widely used to obtain high-quality microbial data and reconstruct the genomes of individual species from environmental communities [1] and human bodies [2]. Many assemblers have been developed for computationally reconstructing microbial communities using sequencing reads [3–6]. Reference-based taxonomic annotation has been used to identify the genomes; however, it was estimated that only 2.1% of prokaryotic genomes have been sequenced [7]. In addition, limited by the complexity of mixed genomes, sequencing bias and errors, the assembled metagenome is still highly fragmented with numerous short contigs. Therefore, the major challenge in metagenomics is how to precisely classify contigs (especially for short contigs) into species-level groups.

To cluster metagenomic contigs, current popular clustering algorithms [8–19] are usually developed on the basis of *k*-mer frequency and coverage, since different microbial genomes have specific composition and abundance. However, the performance of these tools (e.g., CONCOCT [10], VAMB [11], and MetaBAT2 [12]) is relatively poor when dealing with similar genomes. To solve this problem, single-copy marker genes can be added to the model to distinguish similar genomes [13, 14]. However, tools (e.g., MaxBin2 [14]) using marker genes on all contigs directly may not perform well on datasets with high complexity. That is because the number of genomes estimated according to different single-copy marker genes varies in complex datasets, making it difficult to determine the actual number of genomes. To combine the advantages of different tools, an assemble method, DASTool [9], was developed to combine the results of different methods and improve cluster quality. However, it is still a challenge to separate complete and pure genomes from environmental samples.

Considering the real metagenomics data are usually complex and containing similar genomes, we hypothesized that using marker genes after reducing the complexity of the datasets might be helpful for clustering metagenomic contigs. Therefore, we designed a two-layer probabilistic model. We first applied a modified Dirichlet process Gaussian mixture model (DPGMM) on all contigs to generate the preliminary clusters to reduce the complexity. Then, each preliminary cluster with lower complexity than the original data containing all contigs was involved in the second layer to be further clustered, which comprised *k*-mer frequency probabilistic model and coverage model on the basis of single-copy marker genes. As shown in our results, applying MetaDecoder on both simulated and real-world datasets demonstrated that it could generate more complete clusters with lower contamination.

## Results

### An overview of MetaDecoder

**Fig. 1:**
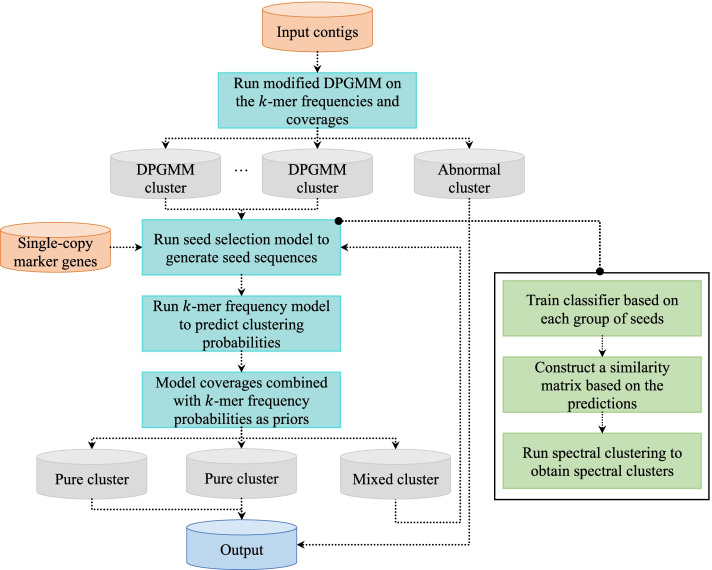
The two-layer architecture of MetaDecoder. A GPU-based modified Dirichlet process Gaussian mixture model (DPGMM) is designed as the first layer to cluster all contigs (≥ 2.5 Kb by default) into preliminary clusters based on the combination of *k*-mer frequency and coverage. These clusters with an average Euclidian distance of pairwise *k*-mer frequencies being greater than 0.04 will be marked as abnormal clusters and removed from the subsequent analysis. Each preliminary cluster is then involved in the second layer to be further clustered, which comprises a semi-supervised *k*-mer frequency probabilistic model with an elaborated seed selection model, and a modified Gaussian mixture model (GMM) as the coverage probabilistic model. Pure clusters (with an estimated genome number of one) are output, and the remaining contigs will continue to the next iteration until all contigs are consumed

MetaDecoder is constructed as a two-layer model (Fig. 1). At the first layer, *k*-mer frequencies and coverages of all contigs are merged together as inputs to the GPU-based modified DPGMM for preliminary clustering, which can dynamically dissolve small clusters and reassign contigs to the remaining clusters to avoid over-segmentation. We monitor the average Euclidian distance of pairwise *k*-mer frequencies within each cluster and discard the abnormal clusters (see the “Methods” section). Each preliminary cluster derived from the DPGMM is involved in the second layer to be further clustered, which comprises two major models modeling *k*-mer frequencies and coverages, respectively. Firstly, semi-supervised *k*-mer frequency probabilistic model is trained by an elaborated seed selection model on the basis of the single-copy marker genes, and then predicts the classification probabilities (Supplementary Fig. S1), which are passed as priors to the coverage probabilistic model. Secondly, a modified multivariate Gaussian mixture model (GMM) models the coverages combined with the adaptive priors to cluster the contigs using the expectation-maximization (EM) algorithm. Clusters with an estimated genome number of one are output. The remaining contigs will continue to the next iteration. The iterations will stop until all contigs are consumed.

### Clustering benchmarks on simulated datasets

**Fig. 2:**
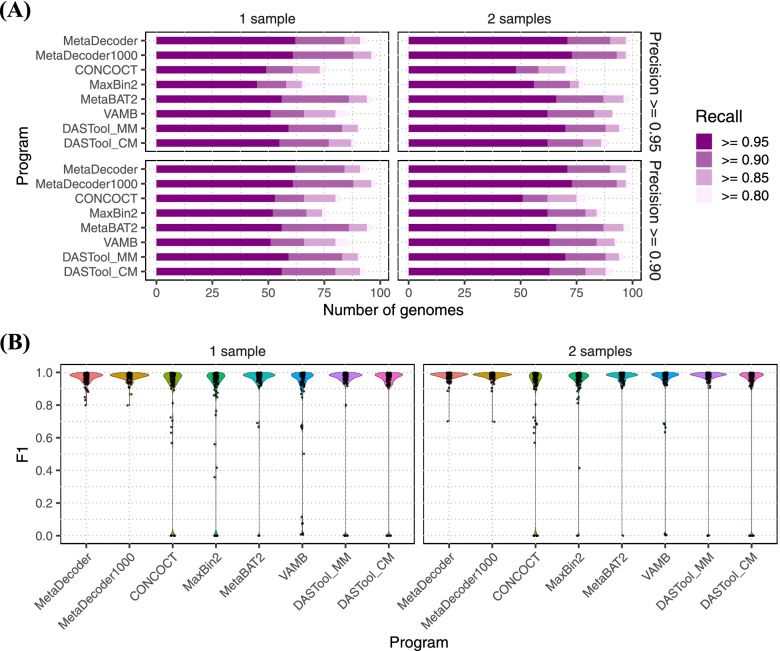
Clustering benchmarks on a simulated dataset. **A** The number of clusters with different recall levels were shown at two precision levels. Programs were run with single or two samples. **B** The F1 scores of all programs run with a single and two samples. All programs were run with their default parameters. DASTool was run with two combinations: (1) DASTool_MM for MetaDecoder and MetaBAT2; (2) DASTool_CM for CONCOCT and MetaBAT2. MetaDecoder with minimum sequence length setting to 1 Kb (MetaDecoder1000) was also added for benchmarking

To evaluate the clustering performance of MetaDecoder on simulated datasets, we first simulated two samples of sequencing reads based on 100 genomes (Supplementary Table S1) and constructed an assembly containing 20,412 contigs. Benchmarks of MetaDecoder and other programs were summarized in Fig. 2, Supplementary Fig. S2, and Supplementary Table S2. The programs included MetaDecoder, MetaDecoder with the minimum sequence length setting to 1 Kb (hereafter referred as MetaDecoder1000), CONCOCT [10], MaxBin2 [14], MetaBAT2 [12], VAMB [11], and two combinations of DASTool [9] (DASTool_MM: MetaDecoder and MetaBAT2; DASTool_CM: CONCOCT and MetaBAT2). Among all programs, MetaDecoder achieved the highest average F1 scores with both single and multiple samples. For the single sample, the average F1 scores of all programs were 96.52% (MetaDecoder), 97.14% (MetaDecoder1000), 85.07% (CONCOCT), 84.43% (MaxBin2), 94.72% (MetaBAT2), 88.49% (VAMB), 89.62% (DASTool_MM), and 90.19% (DASTool_CM), respectively. The numbers of high-quality clusters (*precision* (*purity*) ≥ 0.95 and *recall* (*completeness*) ≥ 0.90) identified by each program were 84 (MetaDecoder), 88 (MetaDecoder1000), 61 (CONCOCT), 58 (MaxBin2), 86 (MetaBAT2), 66 (VAMB), 83 (DASTool_MM), and 77 (DASTool_CM). MetaDecoder and VAMB revealed all 100 genomes in the metagenome, while 10, 10, 2, 7, and 7 genomes were discarded by CONCOCT, MaxBin2, MetaBAT2, DASTool_MM, and DASTool_CM, respectively. There were three similar *Bacillus* species, *B*. *cellulosilyticus*, *B*. *cytotoxicus*, and *B*. *thuringiensis*, of which *B*. *cytotoxicus* was not recognized by any other clustering programs except for MetaDecoder and VAMB. In addition, *B*. *thuringiensis* was missed from the results of MaxBin2, DASTool_MM, and DASTool_CM. The F1 scores of *B*. *thuringiensis* reported by CONCOCT and MetaBAT2 were 63.03% and 69.06%, respectively. Although VAMB found all three *Bacillus* species, it only obtained a very low F1 score (0.52%), while MetaDecoder obtained the highest F1 score of 89.93%. For multiple samples, the average F1 scores of MetaDecoder, MetaDecoder1000, CONCOCT, MaxBin2, MetaBAT2, VAMB, DASTool_MM, and DASTool_CM were 97.49%, 97.54%, 82.63%, 92.79%, 96.45%, 92.53%, 93.95%, and 89.52%, respectively. The numbers of high-quality clusters identified by these programs were 90, 93, 58, 72, 87, 83, 88, and 78, respectively. MetaDecoder and VAMB identified all genomes, while CONCOCT, MaxBin2, MetaBAT2, DASTool_MM, and DASTool_CM missed 12, 3, 1, 4, and 8 genomes, respectively. It was noted that MetaDecoder achieved the highest average precision of 99.84% compared to others (CONCOCT: 82.97%, MaxBin2: 93.82%, MetaBAT2: 98.87%, VAMB: 97.89%, DASTool_MM: 95.88% and DASTool_CM: 91.33%).

### Clustering benchmarks on CAMI datasets

We next benchmarked MetaDecoder and other programs on some more complex Critical Assessment of Metagenome Interpretation (CAMI) datasets (CAMI I Medium and High Complexity datasets, 64 CAMI II Mouse gut datasets and five CAMI II Human Microbiome Project datasets). The CAMI aims to identify and implement best practices for benchmarking in microbiome research (e.g., clustering metagenomic contigs) [20].

**Fig. 3:**
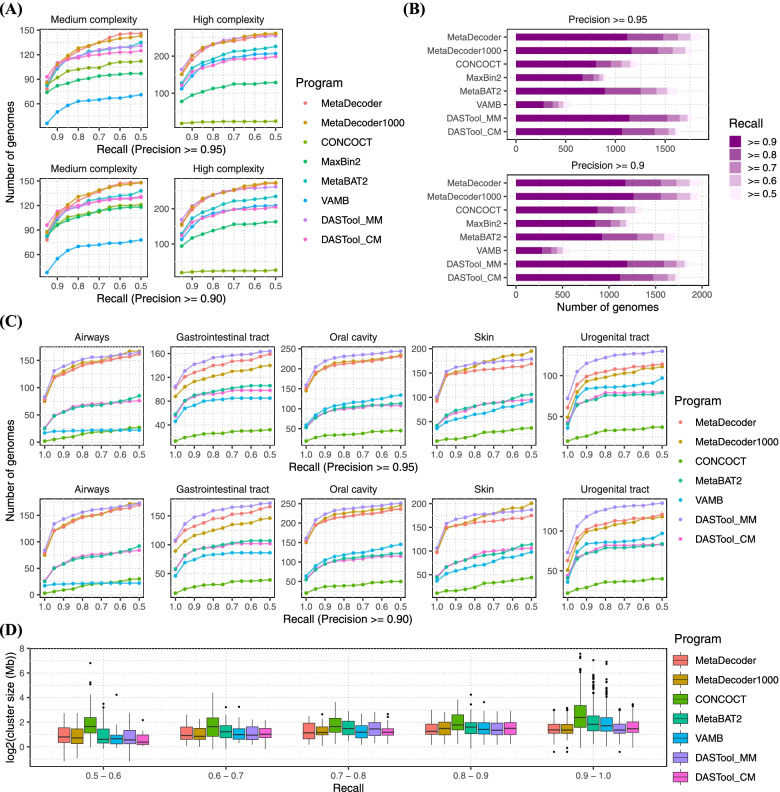
Clustering benchmarks on CAMI datasets. The number of clusters with different recall levels are shown at two precision levels (**A** CAMI I Medium and High Complexity datasets, **B** CAMI II Mouse gut datasets, **C** CAMI II Human Microbiome Project datasets). **D** Distribution of cluster size at each recall level on CAMI II Human Microbiome Project datasets. All programs were run in multi-sample mode (if have multiple samples) with their default parameters except for MaxBin2 on CAMI I High Complexity dataset. MaxBin2 was run with a single merged sample on this dataset and disabled on the CAMI II Human Microbiome Project datasets due to the excessive runtime with multiple samples. DASTool was run with two combinations: (1) DASTool_MM for MetaDecoder and MetaBAT2; (2) DASTool_CM for CONCOCT and MetaBAT2. MetaDecoder with minimum sequence length setting to 1 Kb (MetaDecoder1000) was also added for benchmarking.

In two CAMI I datasets with different complexity (225 and 450 genomes for Medium and High Complexity datasets), MetaDecoder obtained the most high-quality clusters (*precision* ≥ 0.95 and *recall* ≥ 0.90) (Fig. 3A, Supplementary Fig. S3, and Supplementary Table S3). For CAMI I Medium Complexity dataset, the number of high-quality clusters predicted by each program was 108 (MetaDecoder), 108 (MetaDecoder1000), 92 (CONCOCT), 82 (MaxBin2), 103 (MetaBAT2), and 50 (VAMB). MetaDecoder also generated more clusters at each threshold (*recall* from 0.95 to 0.50 under the *precision* threshold of 0.95 or 0.90) on CAMI I High Complexity dataset. There were 190 and 201 high-quality clusters identified by MetaDecoder and MetaDecoder1000 from this complex dataset, while MetaBAT2 (which performs best among the remaining programs) identified 168 clusters with the same threshold. Based on the combination of MetaDecoder and MetaBAT2, DASTool reported 202 high-quality clusters. These results indicate that our program can generate more complete clusters with lower contamination on complex datasets.

In a total of 64 CAMI II Mouse gut datasets (Fig. 3B, Supplementary Figs. S4, S5, and Supplementary Table S4), 804, 669, 895, 277, and 1068 high-quality clusters were predicted by CONCOCT, MaxBin2, MetaBAT2, VAMB, and DASTool_CM, respectively. For comparison, the numbers of high-quality clusters identified by MetaDecoder, MetaDecoder1000, and DASTool_MM were 1,120, 1162 and 1141 respectively. In addition, MetaDecoder also identified a higher number of clusters under each precision and recall threshold.

In five CAMI II Human Microbiome Project datasets, we removed MaxBin2 duo to its excessive runtime with multiple samples. MetaDecoder showed considerable performance at every threshold level on these five datasets (Fig. 3C, Supplementary Fig. S6, and Supplementary Table S5). The largest genome in these five CAMI HMP datasets was 11,694,096 bp. MetaDecoder and DASTool did not report any of the genomes larger than this size (Supplementary Table S5). Meanwhile, CONCOCT, MetaBAT2, and VAMB predicted 99, 46, and 28 clusters larger than this size (Fig. 3D). They may place different genomes in the same cluster, which will be filtered out under *precision* ≥ 0.95. We got an additional improvement using a combination of MetaDecoder and MetaBAT2 with DASTool. It improved the purity of clusters and recovered the most high-quality genomes.

### Clustering benchmarks on real-world datasets

**Fig. 4:**
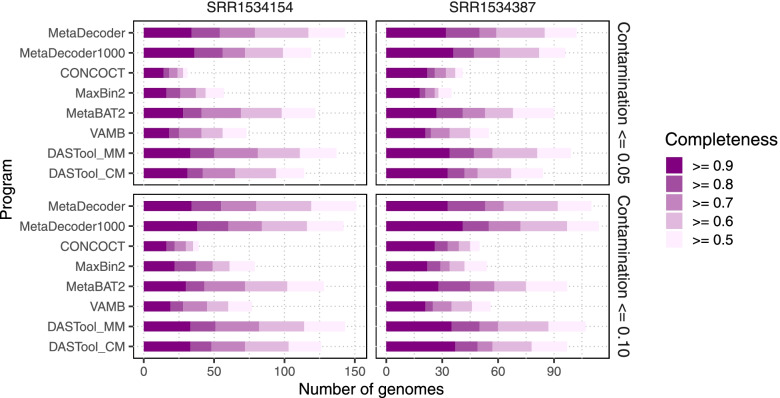
Clustering benchmarks on two datasets from a high-CO_2_ cold-water geyser. The number of clusters with different completeness levels are shown at two contamination levels. All programs were run with their default parameters. DASTool was run with two combinations: (1) DASTool_MM for MetaDecoder and MetaBAT2; (2) DASTool_CM for CONCOCT and MetaBAT2. MetaDecoder with minimum sequence length setting to 1 Kb (MetaDecoder1000) was also added for benchmarking

For the real-world datasets, we first used two environmental datasets from a high-CO_2_ cold-water geyser for benchmarking MetaDecoder (including MetaDecoder1000) and other programs. Two samples (SRR1534387 and SRR1534154) were collected on 3.0 μm and 0.2 μm filters from subsurface aquifers, respectively. We used CheckM [21] to evaluate completeness and contamination of clusters produced by all programs. The results showed that MetaDecoder obtained the most high-quality clusters (contamination ≤ 0.05 and completeness ≥ 0.90) on both datasets (SRR1534387: 32 and SRR1534154: 34), compared to CONCOCT [10] (22 and 14), MaxBin2 [14] (18 and 16), MetaBAT2 [12] (27 and 28), VAMB [11] (21 and 18) (Fig. 4 and Supplementary Table S6). With the combination of MetaDecoder and Metabat2, DASTool recovered 34 and 33 high-quality clusters for SRR1534387 and SRR1534154, respectively. Taxonomic classifications of these 66 high-quality clusters were determined by GTDB-TK [22–24] (Supplementary Table S7). We obtained a new high-quality member (SRR1534154.metadecoder.15) in genus *Sulfurimonas*. Species in this genus are sulfur-oxidizing bacteria and commonly live in deep sea-vents, terrestrial habitats and marine sediments [25].

**Fig. 5:**
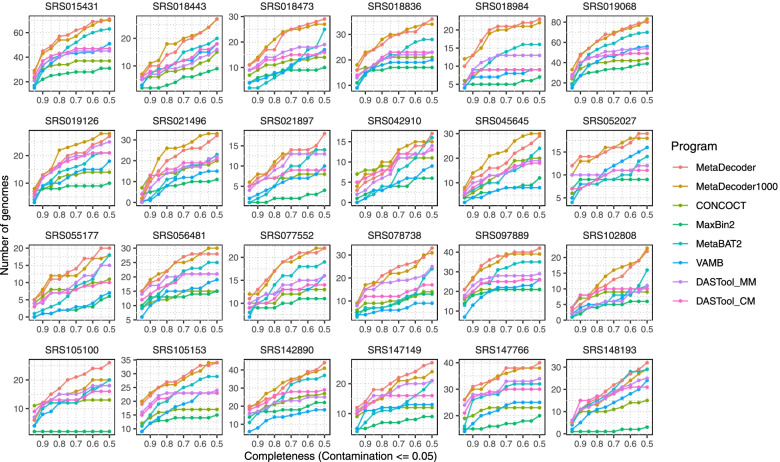
Clustering benchmarks on 24 Human Microbiome Project datasets. The number of identified clusters with *contamination* ≤ 0.05 and different completeness levels are shown. All programs were run with their default parameters. DASTool was run with two combinations: (1) DASTool_MM for MetaDecoder and MetaBAT2; (2) DASTool_CM for CONCOCT and MetaBAT2. MetaDecoder with minimum sequence length setting to 1 Kb (MetaDecoder1000) was also added for benchmarking

We next benchmarked the clustering performance on 24 real-world datasets downloaded from Human Microbiome Project (HMP). MetaDecoder identified 778 genomes with *contamination* ≤ 0.05 and *completeness* ≥ 0.50 compared to CONCOCT (415), MaxBin2 (308), MetaBAT2 (639), VAMB (456), DASTool_MM (539), and DASTool_CM (488). Of which, MetaDecoder recovered 406 high-quality genomes with *completeness* ≥ 0.90 at the same contamination level, while CONCOCT, MaxBin2, MetaBAT2, VAMB, DASTool_MM, and DASTool_CM identified 297, 207, 270, 220, 337, and 344 genomes, respectively (Fig. 5 and Supplementary Table S7). MetaDecoder also showed the best performance when the contamination threshold increased to 0.10 (Supplementary Fig. S7 and Supplementary Table S8). Taxonomic classifications of these clusters showed that we discovered 32 novel high-quality genomes (Supplementary Table S9). For example, “SRS148193.metadecoder.5” (*contamination* ≤ 0.05 and *completeness* ≥ 0.95) was reported as a new member in family *Bacteroidaceae*. Thus, using MetaDecoder, we expanded the reference catalog of human gut bacterial genomes.

### Revealing an increased abundance of Holdemanella species in subjects with impaired glucose tolerance

**Fig. 6.**
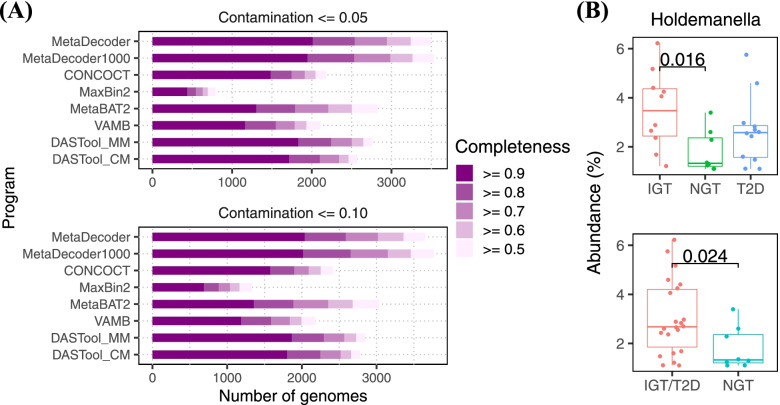
Analyses of T2D dataset. **A** The number of clusters with different completeness levels were shown at two contamination levels. All programs were run with their default parameters. DASTool was run with two combinations: (1) DASTool_MM for MetaDecoder and MetaBAT2; (2) DASTool_CM for CONCOCT and MetaBAT2. MetaDecoder with minimum sequence length setting to 1 Kb (MetaDecoder1000) was also added for benchmarking. **B** Differential abundance of genus *Holdemanella* in T2D (*n* = 53), IGT (*n* = 49), and NGT (control, *n* = 43) samples. Relative abundance was estimated using CheckM and its profile function. Significance was determined using Wilcoxon rank-sum test

We carried out MetaDecoder and all other programs on a cohort of type 2 diabetes (T2D) study containing stool samples from 145 Swedish women, 53 of which were T2D samples [26]. Assessments by CheckM were presented in Fig. 6A; Supplementary Figs. S8, S9; and Supplementary Table S10. MetaDecoder predicted 3651 clusters with contamination ≤ 0.10 and completeness ≥ 0.50, more than the clusters produced by MetaBAT2 (which performs best among the remaining programs) at the same threshold (3036). Further, 2989 clusters were identified with the same taxonomic classification, 662 and 47 clusters were exclusively identified by MetaDecoder and MetaBAT2, respectively. We obtained a total of 2014 high-quality clusters (contamination ≤ 0.05 and completeness ≥ 0.90), compared to MetaBAT2 (1302), DASTool_MM (1865) and DASTool_CM (1802). Taxonomic classifications of 3651 clusters were determined using GTDB-TK [22–24] (Supplementary Table S11). They were classified into an archaeal phylum and six bacterial phyla, with the most frequent phylum being *Firmicutes* (78.58%), suggesting a very broad metabolic potential in the gut (Supplementary Fig. S10). Furthermore, we found a new significant increase in the abundance of *Holdemanella* species in subjects with impaired glucose tolerance (IGT, pre-diabetic states) (Fig. 6B and Supplementary Table S12). *Holdemanella biformis* has been proven to be associated with an unhealthy fasting serum lipid profile [27]. Therefore, generating more complete clusters with MetaDecoder could reveal more disease specific gut metagenomics signatures.

### Benchmarking computing efficiency

We ran all programs on two CAMI I datasets for benchmarking computing efficiency (Table 1 and Supplementary Table S13). MetaDecoder was faster than other tools when analyzing the CAMI I Medium Complexity dataset. For the CAMI I High complexity dataset, MetaDecoder was similar but slightly lower to the fastest program, MetaBAT2. Moreover, the DPGMM layer in MetaDecoder can be accelerated by the GPU to achieve the significant improvement in computational efficiency. MetaDecoder finished clustering in 4 min on the CAMI I High complexity dataset, which is at least 3 times faster than other programs.

**Table 1:** Runtime and memory comparison on CAMI I Medium and High Complexity datasets. All programs were run on a workstation with an Intel(R) Xeon(R) Silver 4108 CPU @ 1.80 GHz (16 threads in total), a NVIDIA Tesla K80 GPU and 96 GB memory. All programs were run with all threads and with their default parameters except for setting the minimum contig length to 2.5 Kb

Runtime memory	MetaDecoder	CONCOCT (version 1.0.0)	MaxBin2 (version 2.2.4)	MetBAT2 (version 2.12.1)	VAMB (version 3.0.2)
CAMI I Medium	5 m 11 s, 0.9 GB 2 min 7 s, 0.9 GB^a^	9 min 7 s, 0.7 GB	133 min 3 s, 0.8 GB	6 min 4 s, 0.8 GB	17 min 34 s, 0.5 GB 8 min 22 s, 0.5 GB^a^
CAMI I High	17 min 38 s, 2.6 GB 4 min 2 s, 2.6 GB^a^	19 min 25 s, 1.1 GB	331 min 8 s, 1.7 GB	13 min 36 s, 1.5 GB	31 min 18 s, 1.7 GB 14 min 0 s, 1.7 GB^a^

Note: ^a^Run with GPU acceleration

### Performance of MetaDecoder and MetaDecoder1000

The default length cutoff for contigs of MetaDecoder was 2.5 Kb, which was the same as MATBAT2. We benchmarked MetaDecoder and MetaDecoder1000 (which uses 1 Kb as the cutoff) on 171 real-world datasets (i.e., two water datasets, 24 HMP datasets and 145 T2D datasets), and no significant difference was detected (Supplementary Fig. S11). To speed up analysis, we recommend the users to use 2.5 Kb as the cutoff. However, users can set their own cutoff with the option of “--min_sequence_length”.

## Discussion

We have introduced MetaDecoder, a new program developed to accurately cluster metagenomic contigs on the basis of the combination of their *k*-mer frequency and coverage. Benchmarks on simulated and real-world datasets demonstrated that MetaDecoder outperformed other programs.

MetaDecoder can produce more complete and pure clusters, which is important to explore the phylogenetic diversity of microbial genomes, particularly for species that are not easily cultured [28–30]. We assessed MetaDecoder’s performance on CAMI benchmarking datasets with the ground truth. MetaDecoder identified the most high-quality clusters (*precision* ≥ 0.95 and *recall* ≥ 0.90) on all datasets as well as under other threshold levels (Fig. 3A–C). For real-world datasets, we reported 33 novel high-quality genomes. These findings expanded the existing catalog of bacterial genomes. For a gut metagenomic dataset of T2D study, we identified 54.69% more high-quality clusters than MetaBAT2 and found a previously reported significant decreased abundance of *Roseburia* species in T2D samples (*p* = 0.033) [31], and a new increased abundance of *Holdemanella* species in IGT samples (Fig. 6B).

The runtime and memory cost of MetaDecoder is comparable to other programs on CPU-based workstations. Furthermore, MetaDecoder is at least three times faster than other current tools when working on GPUs. With the advances in sequencing technologies, the metagenomic data for large scale populations have been generated. For example, as of August 2020, the Integrated Microbial Genomes (IMG) released 26,488 metagenomes and 85,565 high-quality and medium-quality metagenome clusters [32]. The massive number of fragments is one of the most major characteristics of metagenomic data and is also a challenge for clustering. GPU contains massive parallel processors and can perform a large number of matrix calculations very quickly. Therefore, the GPU-based DPGMM in MetaDecoder would offer help for metagenomics researchers in a time efficient manner.

## Conclusions

In conclusion, we developed MetaDecoder for clustering metagenomic contigs and reconstructing microbial communities from microbial data. Applying MetaDecoder on both simulated and real-world datasets demonstrated that it could generate more complete clusters with lower contamination. Moreover, MetaDecoder can be accelerated by the GPU to achieve the significant improvement in computational efficiency. Therefore, we believe that MetaDecoder can be served as a promising approach for effectively clustering metagenomic contigs.

## Methods

### The GPU-based modified Dirichlet process Gaussian mixture model

Given a collection of contigs, we first partition them into preliminary clusters based on their *k*-mer frequencies and coverages. Clustering contigs together is equivalent to assigning them a set of same parameters that describe a distribution, which also means that the distribution of parameters is discretized. With respect to an ambiguous number of clusters *G*, a good choice is to apply the Dirichlet process (DP) to construct a Gaussian mixture model since a draw from DP is a discrete distribution and it is not sensitive to the number of clusters. A DP consists of a base measure ${\mathbbm{G}}_0$ and a concentration parameter *α*, which is expressed as follows:

$$\mathbbm{G}\sim DP\left(\alpha, {\mathbbm{G}}_0\right)$$

where $\mathbbm{G}$ is a random measure. And we use the stick-breaking construction to create DP defined as follows:

$${\beta}_i\sim Beta\left(1,\alpha \right)$$

$${\pi}_j={\beta}_j\prod_{i=1}^{j-1}\left(1-{\beta}_i\right),j=1,2,\dots, \infty$$

from which a DP is derived as:

$$\mathbbm{G}=\sum_{j=1}^{\infty }{\pi}_j{\delta}_{\eta_j}$$

where *β* denotes a random variable from Beta distribution with parameters 1, *α*. ${\delta}_{\eta_j}$ is a point mass probability measure, *η*_*j*_ is a random variable followed ${\mathbbm{G}}_0$ with a weight of *π*_*j*_, which can also be viewed as the parameter of the cluster *j* in mixture model. We then use DP to build the Dirichlet process Gaussian mixture model (DPGMM) with *G* clusters. We define *z*_*c*_ as the hidden variable which indicates the cluster that *x*_*c*_ is from, thus, we have:

$${z}_c\sim Cat\left({\pi}_1,{\pi}_2,\dots, {\pi}_G\right)$$

$${x}_c\sim \mathcal{N}\left({\eta}_{z_c}\right)$$

of which *Cat*(*π*_1_, *π*_2_, …, *π*_*g*_) means the categorical distribution parameterized by *π*_1_, *π*_2_, …, *π*_*g*_ and $\mathcal{N}$ denotes a multivariate Gaussian distribution with parameter ${\eta}_{z_c}$ (*i*.*e*., ${\mu}_{z_c}$ and ${\varSigma}_{z_c}$ in GMM). Under the Bayesian framework, the normal-inverse-Wishart distribution is chosen as the base measure ${\mathbbm{G}}_0$ as its conjugacy to the multivariate Gaussian likelihood with unknown mean and covariance. We have:

$$\mathcal{N}I\mathcal{W}\left(\mu, \varSigma |{\mu}_0,\kappa, \varPsi, v\right)=\mathcal{N}\left(\mu |{\mu}_0,\frac{\varSigma }{\kappa}\right){\mathcal{W}}^{-1}\left(\varSigma |\varPsi, v\right)$$

$$\mathcal{N}\left(\mu |{\mu}_0,\frac{\varSigma }{\kappa}\right)=\frac{\kappa^{\frac{d}{2}}}{{\left(2\pi \right)}^{\frac{d}{2}}{\left|\varSigma \right|}^{\frac{1}{2}}}{\exp}\left(-\frac{\kappa }{2}{\left(\mu -{\mu}_0\right)}^T{\varSigma}^{-1}\left(\mu -{\mu}_0\right)\right)$$

$${\mathcal{W}}^{-1}\left(\varSigma |\varPsi, v\right)=\frac{{\left|\varPsi \right|}^{\frac{v}{2}}}{2^{\frac{vd}{2}}{\varGamma}_d\left(\frac{v}{2}\right)}{\left|\varSigma \right|}^{-\frac{v+d-1}{2}}\mathit{\exp}\left(-\frac{1}{2} tr\left(\varPsi {\varSigma}^{-1}\right)\right)$$

where *d* represents the dimension of each *x*_*c*_, *κ* denotes the precision prior on the mean *μ* distribution, and is set to 10^−4^ to reduce the effect of prior *μ*_0_ (defined as the mean of *X* = {*x*_1_, *x*_2_, …, *x*_*n*_}) of *μ*. *Σ* denotes a *d* × *d* matrix. *Ψ* denotes the prior of *Σ* and is set to the covariance of *X*. *v* is the prior of the number of degrees of freedom on the Wishart distribution and is set to *d*. *Γ*_*d*_ represents the multivariate gamma function and |*M*|, *tr*(*M*) are the determinant and trace of matrix *M*, respectively. We use variational method to approximate the posterior distribution, thus we have:

$${\beta}_g\sim Beta\left({\gamma}_{g,1},{\gamma}_{g,2}\right)$$

$${\mu}_g,{\varSigma}_g\sim \mathcal{N}I\mathcal{W}\left({\mu}_{0,g},{\kappa}_g,{\varPsi}_g,{v}_g\right)$$

$${z}_c\sim Cat\left({r}_{c,1},{r}_{c,2},\dots, {r}_{c,G}\right)$$

In MetaDecoder, we put the weight of each contig on DPGMM, which is defined as ${w}_c=\frac{l_c}{\frac{1}{C}{\sum}_{c=1}^C lc}$, where *l*_*c*_ is the length of contig *c*. Because longer contigs contribute more to the estimation of the parameters.

With respect to the responsibilities *r*, we have:

$${\gamma}_{g,1}=1+\sum_{c=1}^C{w}_c{r}_{c,g}$$

$${\gamma}_{g,2}=\alpha +\sum_{c=1}^C\sum_{g^{\prime }=g+1}^G{w}_c{r}_{c,{g}^{\prime }}$$

$${\kappa}_g=\kappa +\sum_{c=1}^C{w}_c{r}_{c,g}$$

$${\mu}_{0,g}=\frac{1}{\kappa_g}\left(\kappa {\mu}_0+\sum_{c=1}^C{w}_c{r}_{c,g}{x}_c\right)$$

$${\varPsi}_g=\varPsi +\sum_{c=1}^C{w}_c{r}_{c,g}\left({x}_c-{\overline{x}}_g\right){\left({x}_c-{\overline{x}}_g\right)}^T+\frac{\kappa \sum_{c=1}^C{w}_c{r}_{c,g}}{\kappa_g}\left({\overline{x}}_g-{\mu}_0\right){\left({\overline{x}}_g-{\mu}_0\right)}^T$$

$${v}_g=v+\sum_{c=1}^C{w}_c{r}_{c,g}$$

where ${\overline{x}}_g$ denotes the weighted mean of *x* in cluster *g* and is defined as ${\overline{x}}_g=\frac{\sum_{c=1}^C{w}_c{r}_{c,g}{x}_c}{\sum_{c=1}^C{w}_c{r}_{c,g}}$, the concentration parameter *α* is set to 1. And the updating formula of *r*_*c*, *g*_ is:

$${r}_{c,g}=\mathit{\exp}\left(\psi \left({\gamma}_{g,1}\right)-\psi \left({\gamma}_{g,1}+{\gamma}_{g,2}\right)+\sum_{g^{\prime }=1}^{g-1}\left(\psi \left({\gamma}_{g^{\prime },2}\right)-\psi \left({\gamma}_{g^{\prime },1}+{\gamma}_{g^{\prime },2}\right)\right)+\frac{1}{2}\left(\sum_{i=1}^d\psi \left(\frac{v_g+1-i}{2}\right)+ dln2-\mathit{\ln}\left(\left|{\varPsi}_g\right|\right)-\frac{d}{\kappa_g}-{v}_g{\left({x}_c-{\mu}_{0,g}\right)}^T{\varPsi}_g^{-1}\left({x}_c-{\mu}_{0,g}\right)\right)\right)$$

where *ψ* denotes the digamma function, which is the logarithmic derivative of the gamma function.

We perform principal component analysis (PCA) on the *k*-mer frequencies with at least 90% of the variance retained, and then concatenate with the log-transformed coverages as the input to DPGMM. The *k*-mer frequency contributes more to DPGMM since the length of the coverage vector (the number of samples) usually does not exceed the PCA-processed *k*-mer frequency vector. While for contigs with similar *k*-mer frequencies, the length of the *k*-mer frequency vector will be reduced after PCA processing, and therefore, DPGMM can assign more weight to the coverage. This is the so-called intrinsic balance of the two features. DPGMM starts with 3 times value of *G* clusters estimated by the single-copy marker genes (see the seed selection model section), since the effective number can be inferred from the data. We dynamically track the weight of each component with an interval of 10 iterations.

One important point in our modified model is to keep the integrity of each component. Contigs in small components (< 500 Kb by default) will be dissolved and reassigned to the remaining components to avoid over-segmentation, we remove the small component and re-normalize responsibilities as follows:

$${r}_{c,g\ne {g}^{\prime }}=\frac{r_{c,g\ne {g}^{\prime }}}{\sum_{g\ne {g}^{\prime }}{r}_{c,g}}$$

$${r}_c=\left({r}_{c,1},\dots {r}_{c,{g}^{\prime }-1},{r}_{c,{g}^{\prime }+1},\dots {r}_{c,G}\right)$$

where *g*^′^ denotes a small component. As shown in Supplementary Fig. S12, we found that over 98.35% intra-distances are less than or equal to 0.04. In addition, in our simulations data, clusters with average Euclidean distance > 0.04 usually contains many short contigs, which is difficult for classification. Therefore, clusters with k-mer frequency Euclidean distance > 0.04 are considered as abnormal clusters in our first layer. We also tested other values above 0.04 (e.g., 0.05 and 0.1) and the results of our simulated datasets were similar. However, users can use the option “--max_dpgmm_distance” in MetaDecoder to modify this cutoff.

### K-mer frequency probabilistic model

MetaDecoder uses the frequencies of tetramers (*k* = 4) of each contig as signatures, which has been proven to be a successful strategy for microbial genomes discovery [33], and can also support any positive integer as an input parameter. The *k*-mers from the two strands are merged and considered as a kind of *k*-mer.

The semi-supervised *k*-mer frequency probabilistic model uses a group of seeds generated by the seed selection model as the training set (see the seed selection model section). For each seed, 300 sub-seeds (3–4 Kb) are randomly selected. The multi-class SVM with a radial basis function (RBF) kernel is then trained on the *k*-mer frequencies of all sub-contigs with the corresponding labels. The parameter *γ* of the RBF kernel is defined as $\gamma =\frac{1}{\#\mathrm{distinct}\ k- mers}$ (#distinct *k* − *mers* = 136 when *k* = 4). Based on the fitted model, the probability ${p}_{SVM(c)}^g$ that each contig *c* belongs to genome *g* are predicted.

### Coverage probabilistic model

The sequencing coverage reflects the abundance of microorganisms and can therefore be used to determine the source of each contig. There are three main reasons why we designed this model. Firstly, in theory, the coverage follows the Poisson distribution with the parameter *λ* representing the average coverage of the genome; however, the positional coverages are significantly different so the variance may not equal to *λ*. Secondly, each contig has its own *k*-mer frequency probability ${p}_{SVM(c)}^g$, which can be used as a prior. Thirdly, we believe that compared to the *k*-mer frequency probabilistic model, the coverage probabilistic model will become more reliable with increasing number of samples, we need to adjust the effect of the prior on it. Thus, we developed a modified GMM to model the coverages defined as follows:

$$\ell \left(Y;{p}_{SVM},\pi, \mu, \varSigma \right)=\sum_{c=1}^C\mathit{\log}\left(\sum_{g=1}^G{\pi}_c^g\mathcal{N}\left({x}_c|{\mu}^g,{\varSigma}^g\right)\right)-W\sum_{c=1}^C KL\left({p}_{SVM(c)}\Big\Vert {\pi}_c\right)$$

$$c\in \left\{1,2,\dots, C\right\},g\in \left\{1,2,\dots, G\right\}$$

In our model, the first term is the likelihood of the data. Each Gaussian component represents a genome. ${\pi}_c^g$ represents the prior probability that the contig *c* belongs to the genome *g*, and therefore the component weights are data-dependent, which is different from a traditional GMM. *x*_*c*_ indicates the log-transformed coverage of contig *c*. $\mathcal{N}\left({x}_c|{\mu}^g,{\varSigma}^g\right)$ denotes a Gaussian probability density function (PDF) parameterized by the mean vector *μ*^*g*^ ∈ *ℝ*^*n*^ and covariance matrix *Σ*^*g*^ ∈ *ℝ*^*n* × *n*^ of the genome *g*, and the PDF is defined as:

$$\mathcal{N}\left({x}_c|{\mu}^g,{\varSigma}^g\right)=\frac{1}{{\left(2\pi \right)}^{\frac{n}{2}}{\left|{\varSigma}^g\right|}^{\frac{1}{2}}}\mathit{\exp}\left(-\frac{{\left({x}_c-{\mu}^g\right)}^T{\varSigma}^{g-1}\left({x}_c-{\mu}^g\right)}{2}\right)$$

Of which *n* is the number of sequencing samples, |*Σ*^*g*^|, *Σ*^*g* − 1^ represent the determinant and the inverse of the covariance matrix *Σ*^*g*^, respectively.

The second is the regularization term, which is the weighted sum of Kullback-Leibler divergence between the *k*-mer frequency probability ${p}_{SVM(c)}^g$ and the contig dependent prior probability ${\pi}_c^g$ ($KL\left({p}_{SVM(c)}\Big\Vert {\pi}_c\right)=\sum_{g=1}^G{p}_{SVM(c)}^g\mathit{\log}\left(\frac{p_{SVM(c)}^g}{\pi_c^g}\right)$). *W* is a positive constant in the regularization term and is set to $W=\frac{1}{n}$ by default. The role of this term is to force ${\pi}_c^g$ to be close to ${p}_{SVM(c)}^g$. A small *W* (e.g., a large number of sequencing samples) will weaken this force and therefore enhance the weight of this model. The regularization term will be minimized to zero if the two probabilities are the same. It indicates that if two probabilistic models make the same decision for the same contig, the likelihood will be maximized. The EM algorithm is used to solve this problem, which contains two main steps as follows:

E-step:

$${r}_c^g=\frac{\pi_c^g\mathcal{N}\left({x}_c|{\mu}^g,{\varSigma}^g\right)}{\sum_{g=1}^G{\pi}_c^g\mathcal{N}\left({x}_c|{\mu}^g,{\varSigma}^g\right)}$$

where ${r}_c^g$ is the posterior probability (responsibility) that the contig *c* belongs to the genome *g*.

M-step:

$${\pi}_c^g=\frac{r_c^g+W{p}_{SVM(c)}^g}{1+W}$$

$${\mu}^g=\frac{\sum_{c=1}^C{r}_c^g{x}_c}{\sum_{c=1}^C{r}_c^g}$$

$${\varSigma}^g=\frac{\sum_{c=1}^C{r}_c^g{\left({x}_c-{\mu}^g\right)}^T\left({x}_c-{\mu}^g\right)}{\sum_{c=1}^C{r}_c^g}$$

Before running the EM algorithm, some parameters such as *μ*^*g*^, *Σ*^*g*^, and ${\pi}_c^g$ must be determined, which usually be randomly initialized; however, this might cause the EM algorithm to fall into a locally optimal solution. Therefore, we take the *k*-mer frequency probability ${p}_{SVM(c)}^g$ as the prior ${\pi}_c^g$ in the EM algorithm and then to initialize all necessary parameters. The E-step and M-step are repeated until convergence. Clusters (> 200 Kb by default) generated from this model with an estimated genome number of one are output. Therefore, there will be no clusters smaller than 200 Kb with default parameters. However, users can change this cutoff by using the “--min_cluster_size” option. Contigs in abnormal clusters from the first layer or in small clusters (i.e., < 200 Kb) from this model are defined as the unclustered contigs. Users could use the “--output_unclustered_sequences” option to output all unclustered contigs.

### Seed selection model

**Fig. 7:**
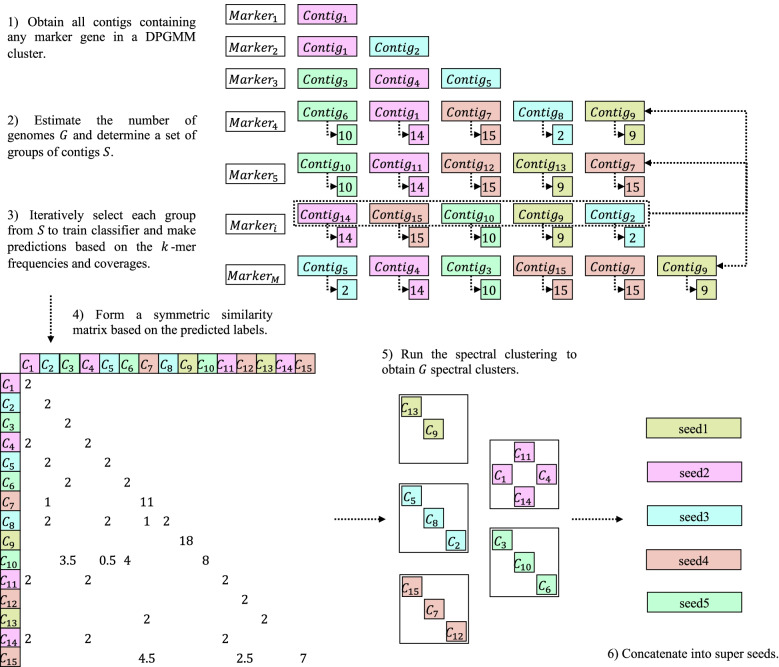
Framework of seed selection model. Assume that a set of *M* single-copy marker genes are mapped to 15 contigs with hidden origins indicated by different colors, we first estimate the number of genomes as *G* = 5 and determine a set ($\mathcal{S}$) of groups of contigs containing marker genes {*m*_4_, …, *m*_*M*_}. Then the classifier is trained with each group of contigs from $\mathcal{S}$ and predicts all contigs in $\mathcal{S}$ based on both *k*-mer frequencies and coverages to form a symmetric similarity matrix. We next run spectral clustering algorithm to obtain spectral clusters. And finally, contigs in each spectral cluster are concatenated into a single extended seed

The *k*-mer frequency probabilistic model in MetaDecoder is a semi-supervised model with training set provided by this model (Fig. 7). Assume that there are several single-copy marker genes harbored in a DPGMM cluster, and one of them, *m*_*i*_, is mapped to *G*_*i*_ contigs. Ideally, *m*_*i*_ is single-copy and shared among all genomes, all contigs are long enough with no assembly errors, then with respect to *m*_*i*_, the number of genomes in this dataset should be estimated as *G*_*i*_. However, there are many restrictions in practice. Due to limited mapping method, sequencing errors, or poor assembly quality, *m*_*i*_ may be lost or multiplied in real-world datasets, resulting in different numbers of contigs {*G*_1_, *G*_2_, …, *G*_*i*_} containing the marker genes {*m*_1_, *m*_2_, …, *m*_*i*_}. Intuitively, the estimated number of genomes should be greater than or equal to the mode of {*G*_1_, *G*_2_, …, *G*_*i*_} (*G*_*mode*_) and thus can be roughly determined as follows:

$$G=\max \left({G}_i\ if\#{G}_i\ge \#{G}_{\mathrm{mode}}\times 0.5\right)$$

where #*G*_*i*_, #*G*_mode_ are the counts of *G*_*i*_ and *G*_mode_ in the set {*G*_1_, *G*_2_, …, *G*_*i*_}, respectively. We collect a set of groups of contigs $\mathcal{S}=\left\{{\mathbbm{g}}_1,{\mathbbm{g}}_2,..,{\mathbbm{g}}_S\right\}$ with *G*_*s*_ ≥ *G*_mode_. Contigs in each $\mathbbm{g}$ are more likely to derive from different genomes, since they contain the same single-copy marker gene. We next need to identify contigs from the same genome but scattered in different $\mathbbm{g}$. An adjacency matrix *A* is defined to measure the possibility that any two contigs belong to the same genome. For each ${\mathbbm{g}}_s\in \mathcal{S}$, 20 sub-contigs (1.5–2.5 Kb) are randomly sampled from each contig to train the multi-class SVM, then all contigs are predicted. The possibility that each pair of contigs (*i*, *j*) belongs to the same genome is measured as follows:

$${A}_{i,j}=\sum_{{\mathbbm{g}}_i,{\mathbbm{g}}_j}\left\{\begin{array}{c}1/\sum_{k\in {\mathbbm{g}}_j}{1}_{\left\{f(k)=i\right\}}, if\ f(j)=i\\ {}0,\mathrm{otherwise}\end{array}\right.$$

where ${\mathbbm{g}}_i$ denotes a group containing contig *i* which is used to train the classifier and ${\mathbbm{g}}_j$ denotes a predicted group containing contig *j*. Function *f*(*j*) is expressed as the predicted label of the contig *j*. *A*_*i*, *j*_ is the *i*th row and *j*th column of the adjacency matrix *A*. It is reasonable to use this matrix to quantify the possibility that contigs should be clustered together. Consider two groups: ${\mathbbm{g}}_i$ is used to train the classifier and ${\mathbbm{g}}_j$ for predicting. *j* will be labeled *i* if they are derived from the same genome, which means that *A*_*i*, *j*_ will obtain a bonus of 1. In some complicated cases, such as $j,k\in {\mathbbm{g}}_j$ and *f*(*j*) = *f*(*k*) = *i*, then *A*_*i*, *j*_ will get a lower bonus of 0.5 since we cannot determine whether *i* and *j* are from the same genome. For reasons of symmetry, *A* plus *A*^*T*^ and the number of clusters *G* are as inputs to run the spectral clustering algorithm. Contigs in each spectral cluster are concatenated into a single extended seed.

### Mapping single-copy marker genes to contigs

Protein coding genes recognized by FragGeneScan (version 1.31) [34] with default parameters were obtained to search against the database formed by the 107 single-copy marker genes shared within 95% of the sequenced bacteria [35] using HMMER [36] (version 3.2.1). Valid hits (coverage ≥ 0.5 and accuracy ≥ 0.6) were preserved for the subsequent analyses. These two programs have been included in MetaDecoder.

### Assemblies constructing and sequencing reads aligning

For our simulated and all real-word datasets, the sequencing data were first de novo assembled into a set of contigs (assembly) using IDBA-UD (version 1.13) [3] with default parameters. For CAMI I and CAMI II datasets, we directly used the pooled gold standard assemblies.

Reads of two samples from high-CO_2_ cold-water geyser were trimmed using sickle (version 1.33) (https://github.com/najoshi/sickle/) with default parameters. Read mapping for all samples was done using Bowtie2 (version 2.3.4.3) [37] with default parameters. For all datasets with multiple samples, we aligned each sample data to the assembly. MetaDecoder used uniquely aligned reads with *MAPQ* ≥ 20 to calculate the coverages of contigs, which is defined as the mean number of bases of a contig.

### Programs and datasets used for benchmarking

In this study, MetaDecoder, CONCOCT (version 1.0.0), MaxBin2 (version 2.2.4), MetaBAT2 (version 2.12.1), VAMB (version 3.0.2), and DASTool (version 1.1.2) with two combinations: 1) DASTool_MM for MetaDecoder and MetaBAT2; 2) DASTool_CM for CONCOCT and MetaBAT2 with the default parameters were applied to reconstruct the genome clusters. The minimum length threshold of contigs of MetaDecoder is set to 2.5 Kb by default, which is the same as the cutoff of MetaBAT2. Since this threshold of CONCOCT and MaxBin2 is 1 Kb, we also provided the performance of MetaDecoder with 1Kb as the cutoff (MetaDecoder1000) for benchmarking. For datasets with multiple samples, we ran them in multi-sample mode except for MaxBin2 on CAMI I High Complexity dataset. We carried out MaxBin2 on a single merged sample duo to its excessive runtime with multiple samples.

To benchmark the semi-supervised *k*-mer frequency probabilistic model, a set of 16,199 random fragments (1–50 Kb) was simulated from 100 sequenced completed bacterial genomes collected from NCBI (Supplementary Table S1).

We used MetaSim (version 0.9.5) [38] to simulate two sets of shotgun sequencing reads based on 100 genomes with random coverages from 10× to 40× using the default parameters and the empirical error model downloaded from http://ab.inf.uni-tuebingen.de/software/metasim/errormodel-80bp.mconf (Supplementary Table S2). Of which, one sample was used to construct a metagenome containing 20,412 contigs.

Simulated short-read CAMI datasets were downloaded from https://data.cami-challenge.org/participate/. For benchmarking on read-world datasets, we downloaded 24 HMP datasets from HMP portal, two datasets from a high-CO_2_ cold-water geyser and a cohort of T2D study which consists of 145 samples from NCBI.

### Clustering benchmarks on simulated and real-world datasets

For CAMI I and CAMI II benchmarking datasets, we used the gold standard mapping files provided in the datasets as input to AMBER (version 2.0.2) [39]. For our simulated dataset, each contig was matched against the reference using BLAST (version 2.7.1) [40] with default parameters and was labeled genome identifier with the highest score. Then we converted the mappings to the gold standard file which can be used by AMBER. Clustering quality was evaluated with AMBER using default parameters to obtain the quality metrics. For real-world datasets, as no reference genomes can be provided, we ran CheckM (version 1.0.13) [21] based on the presence of lineage-specific marker genes with default parameters to evaluate completeness and contamination of each cluster.

### Taxonomic, phylogenetic, and differential abundance analyses of identified clusters

Taxonomic classifications of identified clusters were assigned by GTDB (version 95) using GTDB-TK (version 1.3.0) with default parameters on the basis of the average nucleotide identity (ANI) [22–24]. Novel high-quality genomes (contamination ≤ 0.05 and completeness ≥ 0.95) were determined if the ANI between them and all representative genomes in GTDB was lower than 95%.

For phylogenetic analysis, FastTree (version 2.1.11) [41] with default parameters was used to construct the tree based on the concatenated marker gene amino acid alignments created by CheckM (version 1.0.13) [21]. The phylogenetic tree was visualized in iTOL [42].

Relative abundance was determined as the percentage of reads mapped to each cluster using CheckM and its profile function. Significance was determined using Wilcoxon rank-sum test.

## Supplementary Information

**Additional file 1: Supplementary Figure S1**. Clustering performance of the k-mer frequency probabilistic model. (*A*) PCA of 16,199 fragmented contigs with transparency representing the length and color indicating the genome. (*B*) Clustering performance of the multi-class SVM on all 16,199 contigs. The classifier was trained 100 times with a random region of 50 Kb in length of each genome. Among the 1,619,900 predictions, 92.85% (1,504,091) were accurate. The mean and standard deviation of the clustering probabilities were 0.86±0.17 (correct predictions) and 0.40±0.21 (incorrect predictions), respectively. Genome identifiers were provided in Supplemental Table S1. (*C*) Comparison of clustering probabilities between correct and incorrect predictions. Misclassified predictions have a higher average Shannon entropy (2.35) than the correct predictions (0.71).

**Additional file 2: Supplementary Figure S2**. Clustering benchmarks on a simulated dataset. The number of identified bins with different score levels were shown. All programs were run with their default parameters in both single-sample (A) and multi-sample (B) modes. MetaDecoder with minimum sequence length setting to 1 Kb (MetaDecoder1000) was also added for benchmarking. Assessments were evaluated using AMBER (version 2.0.2).

**Additional file 3: Supplementary Figure S3**. Clustering benchmarks on two CAMI I datasets. The number of identified bins on CAMI I (*A*) Medium and (*B*) High Complexity dataset with different score levels were shown. All programs were run in multi-sample mode with their default parameters except for MaxBin2 on the CAMI I High Complexity dataset. MaxBin2 was run on a single merged dataset duo to the excessive runtime with multiple samples. MetaDecoder with minimum sequence length setting to 1 Kb (MetaDecoder1000) was also added for benchmarking. Assessments were evaluated using AMBER (version 2.0.2).

**Additional file 4: Supplementary Figure S4**. Clustering benchmarks on 64 two CAMI II Mouse gut datasets. The number of identified clusters with precision ≥ 0.95 and different recall levels were shown. All programs were run with their default parameters. MetaDecoder with minimum sequence length setting to 1 Kb (MetaDecoder1000) was also added for benchmarking. Assessments were evaluated using AMBER (version 2.0.2).

**Additional file 5: Supplementary Figure S5**. Clustering benchmarks on 64 two CAMI II Mouse gut datasets. The number of identified clusters with precision ≥ 0.90 and different recall levels were shown. All programs were run with their default parameters. MetaDecoder with minimum sequence length setting to 1 Kb (MetaDecoder1000) was also added for benchmarking. Assessments were evaluated using AMBER (version 2.0.2).

**Additional file 6: Supplementary Figure S6**. Clustering benchmarks on five CAMI II Human Microbiome Project datasets. The number of identified bins on (*A*) Airways, (*B*) Gastrointestinal tract, (*C*) Oral cavity, (*D*) Skin and (*E*) Urogenital tract dataset with different score levels were shown. All programs were run in multi-sample mode with their default parameters. MetaDecoder with minimum sequence length setting to 1 Kb (MetaDecoder1000) was also added for benchmarking. DASTool was carried out on two combinations: 1) MetaDecoder and MetaBAT2, 2) CONCOCT and MetaBAT2. Assessments were evaluated using AMBER (version 2.0.2).

**Additional file 7: Supplementary Figure S7**. Clustering benchmarks on 24 Human Microbiome Project datasets. The number of identified clusters with contamination ≤ 0.10 and different recall levels were shown. All programs were run with their default parameters. MetaDecoder with minimum sequence length setting to 1 Kb (MetaDecoder1000) was also added for benchmarking. Assessments were evaluated using CheckM (version 1.0.13).

**Additional file 8: Supplementary Figure S8**. Clustering benchmarks on a cohort of T2D study. The number of identified clusters with contamination ≤ 0.05 and different recall levels were shown. All programs were run with their default parameters. MetaDecoder with minimum sequence length setting to 1 Kb (MetaDecoder1000) was also added for benchmarking. Assessments were evaluated using CheckM (version 1.0.13).

**Additional file 9: Supplementary Figure S9**. Clustering benchmarks on a cohort of T2D study. The number of identified clusters with contamination ≤ 0.10 and different recall levels were shown. All programs were run with their default parameters. MetaDecoder with minimum sequence length setting to 1 Kb (MetaDecoder1000) was also added for benchmarking. Assessments were evaluated using CheckM (version 1.0.13).

**Additional file 10: Supplementary Figure S10**. Phylogenetic analysis of 3,651 clusters with contamination ≤ 0.10 and completeness ≥ 0.50. Taxonomic labels were assigned by GTDB (version 95) using GTDB-TK (version 1.3.0) with default parameters. FastTree (version 2.1.11) with default parameters was used to construct the tree based on the concatenated marker gene amino acid alignments created by CheckM (version 1.0.13). The tree was visualized in iTOL. Only the bacterial phyla were shown.

**Additional file 11: Supplementary Figure S11**. Clustering benchmarks of MetaDecoder under different contig length thresholds. MetaDecoder with default length threshold (2.5 Kb) and 1 Kb (MetaDecoder1000) were applied on all real-world samples (*i.e.,* two samples from high-CO2 cold-water geyser, 24 HMP samples and 145 samples from a cohort of T2D study), respectively. Cluster number (completeness ≥ 0.90) of each sample was shown at two contamination levels. Significance was determined using the Wilcoxon matched-pairs signed rank test.

**Additional file 12: Supplementary Figure S12**. Euclidean distance distributions between contigs abstracted from the same genome (intra-genome) and from two different genomes (inter-genome). The 3,023 bacterial genomes downloaded from NCBI were randomly fragmented into contigs (1 Kb - 100 Kb), then one million simulations were performed. Two histograms denote the intra- and interdistances, respectively. Two curves represent Gaussian densities parametrized by the mean and variance of the intra- and inter- distances, respectively.

**Additional file 13: Supplementary Table S1**. Information of 100 genomes collected from NCBI.

**Additional file 14: Supplementary Table S2**. Clustering benchmarks on a simulated dataset (20,412 contigs).

**Additional file 15: Supplementary Table S3**. Clustering benchmarks on CAMI I Medium and High Complexity datasets.

**Additional file 16: Supplementary Table S4**. Clustering benchmarks on CAMI II Mouse gut datasets.

**Additional file 17: Supplementary Table S5**. Clustering benchmarks on five CAMI II Human Microbiome Project datasets.

**Additional file 18: Supplementary Table S6**. Clustering benchmarks on two datasets from a high-CO2 cold-water geyser.

**Additional file 19: Supplementary Table S7**. Taxonomic classifications of clusters identified by MetaDecoder with contamination <= 0.10 and completeness >= 0.50 on two datasets from a high-CO2 cold-water geyser.

**Additional file 20: Supplementary Table S8**. Clustering benchmarks on 24 Human Microbiome Project datasets.

**Additional file 21: Supplementary Table S9**. Taxonomic classifications of clusters identified by MetaDecoder with contamination <= 0.10 and completeness >= 0.50 on 24 hmp datasets.

**Additional file 22: Supplementary Table S10**. Clustering benchmarks on a cohort of T2D study.

**Additional file 23: Supplementary Table S11**. Taxonomic classifications of clusters with contamination <= 0.10 and completeness >= 0.50.

**Additional file 24: Supplementary Table S12**. Analysis of relative abundance in IGT, IGT and T2D samples.

**Additional file 25: Supplementary Table S13**. Runtime and memory comparison on CAMI I Medium and High Complexity datasets.

## Data Availability

MetaDecoder is open source and freely available in the GitHub repository: https://github.com/liu-congcong/MetaDecoder/. The simulated CAMI I Medium, High Complexity datasets, CAMI II Mouse gut, and CAMI II Human Microbiome Project datasets were downloaded from https://data.cami-challenge.org/participate/. All reference genomes mentioned in the text are publicly available in the NCBI. The 24 HMP datasets (SRS015431, SRS018443, SRS018473, SRS018836, SRS018984, SRS019068, SRS019126, SRS021496, SRS021897, SRS042910, SRS045645, SRS052027, SRS055177, SRS056481, SRS077552, SRS078738, SRS097889, SRS102808, SRS105100, SRS105153, SRS142890, SRS147149, SRS147766, and SRS148193) are available at HMP portal, two datasets from a high-CO_2_ cold-water geyser and a cohort of T2D study are available in the NCBI with SRA accessions SRP045164 and ERP002469, respectively. Pipelines and benchmarks for all data processing are available in the GitHub repository (https://github.com/liu-congcong/MetaDecoder/), and the processed data are available in the Google Drive (https://drive.google.com/drive/folders/1_mybcewf3VE-7dte6oA-vDmlRx2ugzyD?usp=sharing).
