# Supplementary material for: MetaDecoder: a novel method for clustering metagenomic contigs
Source: Microbiome. 2022 Mar 10;10:46. doi: 10.1186/s40168-022-01237-8 (PMC8908641; doi:10.1186/s40168-022-01237-8)
Supplement: Supplementary file 2 — Additional file 1: Supplementary Figure S1. Clustering performance of the k-mer frequency probabilistic model. (A) PCA of 16,199 fragmented contigs with transparency representing the length and color indicating the genome. (B) Clustering performance of the multi-class SVM on all 16,199 contigs. The classifier was trained 100 times with a random region of 50 Kb in length of each genome. Among the 1,619,900 predictions, 92.85% (1,504,091) were accurate. The mean and standard deviation of the clustering probabilities were 0.86±0.17 (correct predictions) and 0.40±0.21 (incorrect predictions), respectively. Genome identifiers were provided in Supplemental Table S1. (C) Comparison of clustering probabilities between correct and incorrect predictions. Misclassified predictions have a higher average Shannon entropy (2.35) than the correct predictions (0.71). [file 40168_2022_1237_MOESM1_ESM.pdf]

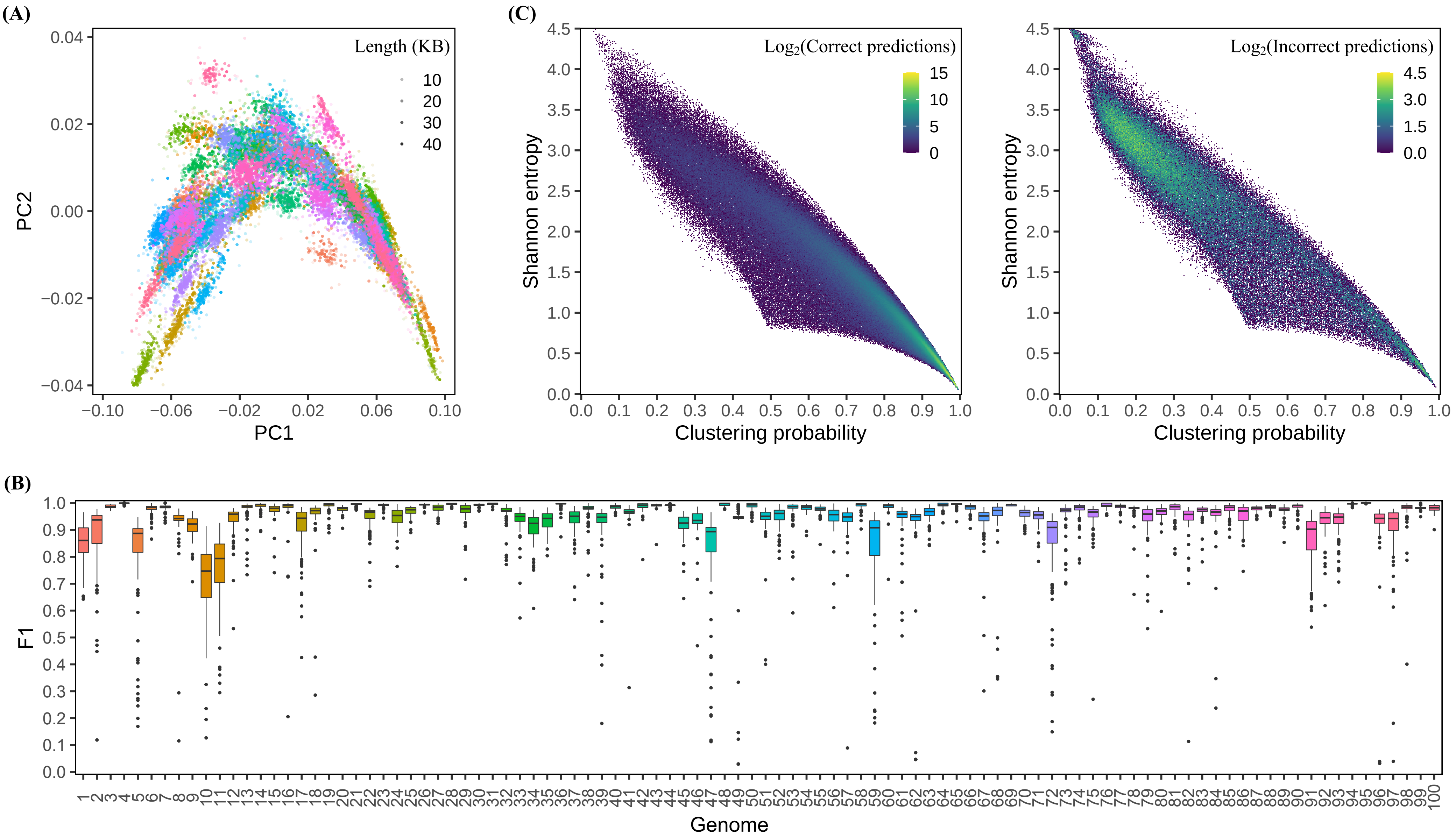

Supplementary Figure S1. Clustering performance of the k-mer frequency probabilistic model. (*A*) PCA of 16,199 fragmented contigs with transparency representing the length and color indicating the genome. (*B*) Clustering performance of the multi-class SVM on all 16,199 contigs. The classifier was trained 100 times with a random region of 50 Kb in length of each genome. Among the 1,619,900 predictions, 92.85% (1,504,091) were accurate. The mean and standard deviation of the clustering probabilities were 0.86±0.17 (correct predictions) and 0.40±0.21 (incorrect predictions), respectively. Genome identifiers were provided in Supplemental Table S1. (*C*) Comparison of clustering probabilities between correct and incorrect predictions. Misclassified predictions have a higher average Shannon entropy (2.35) than the correct predictions (0.71).
