# Supplementary material for: MetaDecoder: a novel method for clustering metagenomic contigs
Source: Microbiome. 2022 Mar 10;10:46. doi: 10.1186/s40168-022-01237-8 (PMC8908641; doi:10.1186/s40168-022-01237-8)
Supplement: Supplementary file 3 — Additional file 2: Supplementary Figure S2. Clustering benchmarks on a simulated dataset. The number of identified bins with different score levels were shown. All programs were run with their default parameters in both single-sample (A) and multi-sample (B) modes. MetaDecoder with minimum sequence length setting to 1 Kb (MetaDecoder1000) was also added for benchmarking. Assessments were evaluated using AMBER (version 2.0.2). [file 40168_2022_1237_MOESM2_ESM.pdf]

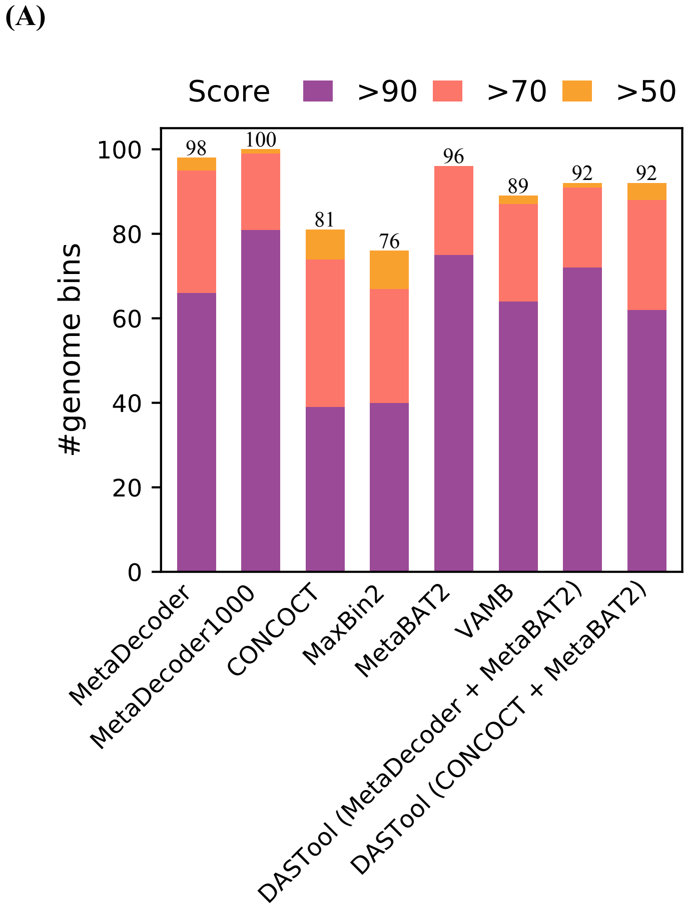

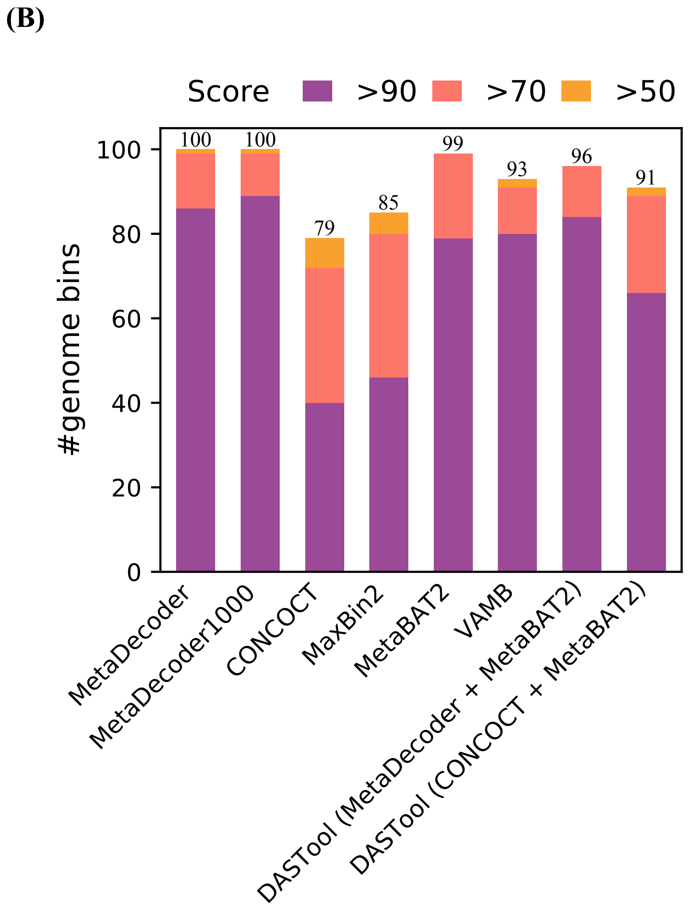

Supplementary Figure S2. Clustering benchmarks on a simulated dataset. The number of identified bins with different score levels were shown. All programs were run with their default parameters in both single-sample **(A)** and multi-sample **(B)** modes. MetaDecoder with minimum sequence length setting to 1 Kb (MetaDecoder1000) was also added for benchmarking. Assessments were evaluated using AMBER (version 2.0.2).
