# Supplementary material for: MetaDecoder: a novel method for clustering metagenomic contigs
Source: Microbiome. 2022 Mar 10;10:46. doi: 10.1186/s40168-022-01237-8 (PMC8908641; doi:10.1186/s40168-022-01237-8)
Supplement: Supplementary file 4 — Additional file 3: Supplementary Figure S3. Clustering benchmarks on two CAMI I datasets. The number of identified bins on CAMI I (A) Medium and (B) High Complexity dataset with different score levels were shown. All programs were run in multi-sample mode with their default parameters except for MaxBin2 on the CAMI I High Complexity dataset. MaxBin2 was run on a single merged dataset duo to the excessive runtime with multiple samples. MetaDecoder with minimum sequence length setting to 1 Kb (MetaDecoder1000) was also added for benchmarking. Assessments were evaluated using AMBER (version 2.0.2). [file 40168_2022_1237_MOESM3_ESM.pdf]

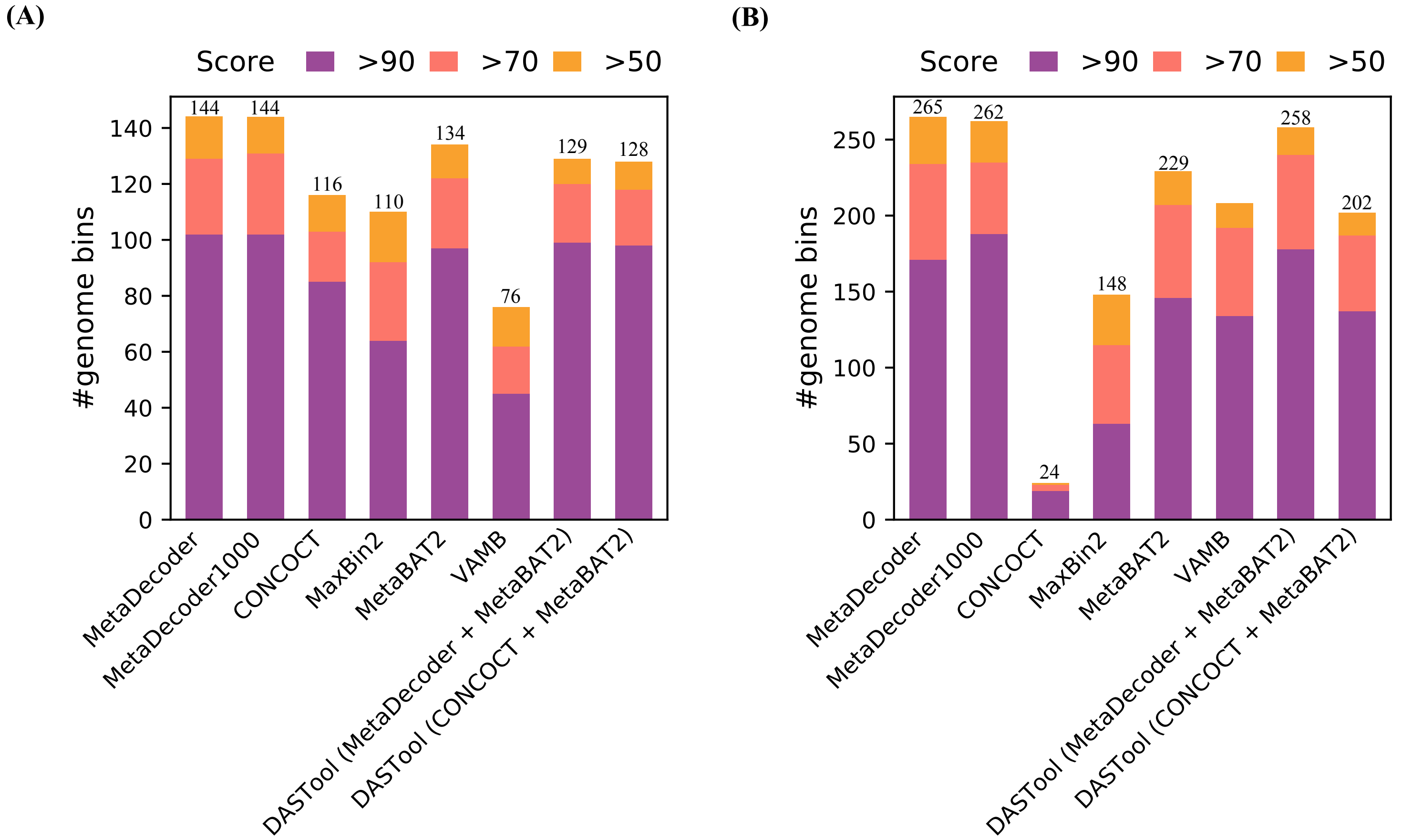

Supplementary Figure S3. Clustering benchmarks on two CAMI I datasets. The number of identified bins on CAMI I (*A*) Medium and (*B*) High Complexity dataset with different score levels were shown. All programs were run in multi-sample mode with their default parameters except for MaxBin2 on the CAMI I High Complexity dataset. MaxBin2 was run on a single merged dataset duo to the excessive runtime with multiple samples. MetaDecoder with minimum sequence length setting to 1 Kb (MetaDecoder1000) was also added for benchmarking. Assessments were evaluated using AMBER (version 2.0.2).
