# Supplementary material for: MetaDecoder: a novel method for clustering metagenomic contigs
Source: Microbiome. 2022 Mar 10;10:46. doi: 10.1186/s40168-022-01237-8 (PMC8908641; doi:10.1186/s40168-022-01237-8)
Supplement: Supplementary file 5 — Additional file 4: Supplementary Figure S4. Clustering benchmarks on 64 two CAMI II Mouse gut datasets. The number of identified clusters with precision ≥ 0.95 and different recall levels were shown. All programs were run with their default parameters. MetaDecoder with minimum sequence length setting to 1 Kb (MetaDecoder1000) was also added for benchmarking. Assessments were evaluated using AMBER (version 2.0.2). [file 40168_2022_1237_MOESM4_ESM.pdf]

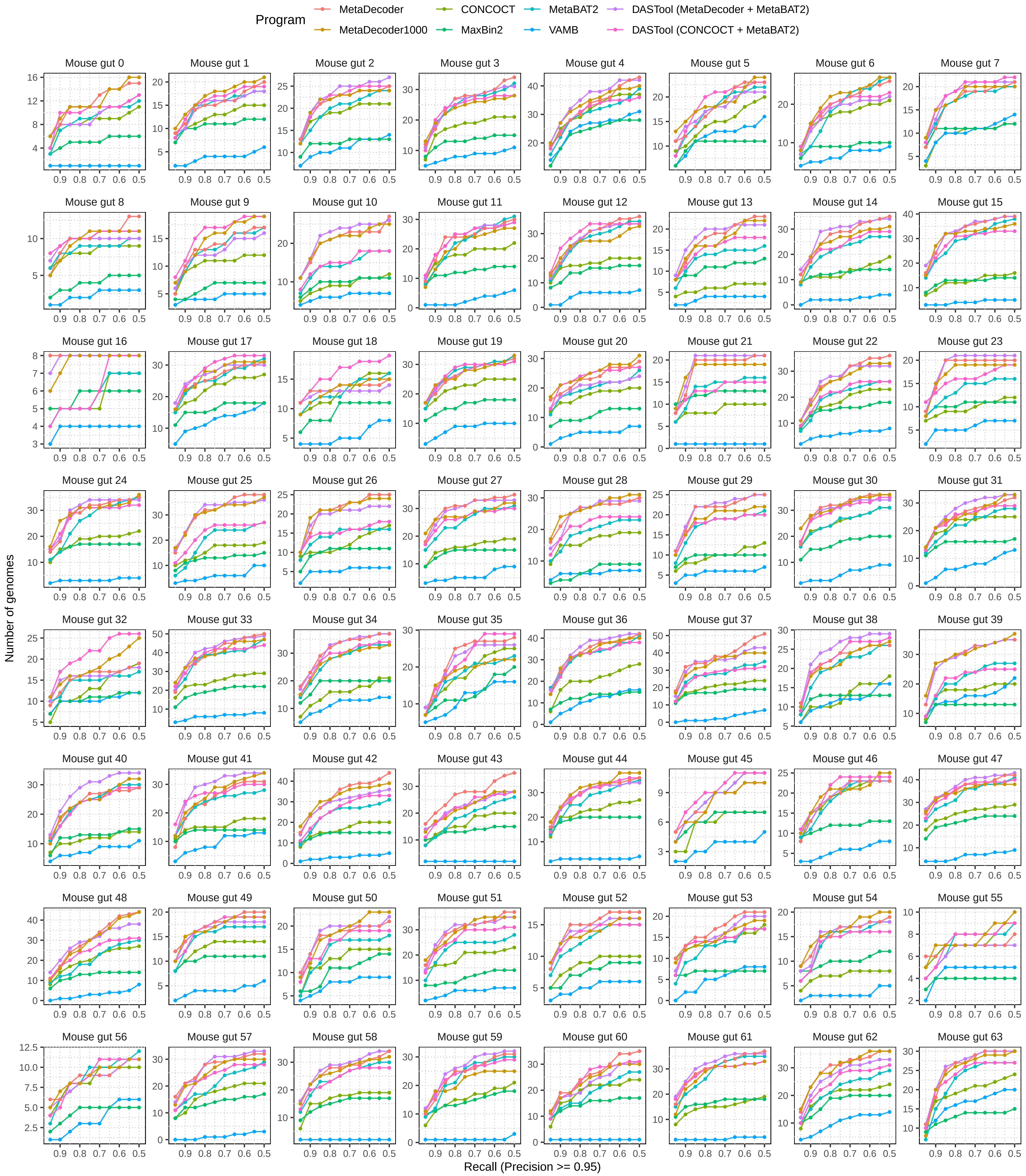

Supplementary Figure S4. Clustering benchmarks on 64 two CAMI II Mouse gut datasets. The number of identified clusters with precision ≥ 0.95 and different recall levels were shown. All programs were run with their default parameters. MetaDecoder with minimum sequence length setting to 1 Kb (MetaDecoder1000) was also added for benchmarking. Assessments were evaluated using AMBER (version 2.0.2).
