# Supplementary material for: MetaDecoder: a novel method for clustering metagenomic contigs
Source: Microbiome. 2022 Mar 10;10:46. doi: 10.1186/s40168-022-01237-8 (PMC8908641; doi:10.1186/s40168-022-01237-8)
Supplement: Supplementary file 7 — Additional file 6: Supplementary Figure S6. Clustering benchmarks on five CAMI II Human Microbiome Project datasets. The number of identified bins on (A) Airways, (B) Gastrointestinal tract, (C) Oral cavity, (D) Skin and (E) Urogenital tract dataset with different score levels were shown. All programs were run in multi-sample mode with their default parameters. MetaDecoder with minimum sequence length setting to 1 Kb (MetaDecoder1000) was also added for benchmarking. DASTool was carried out on two combinations: 1) MetaDecoder and MetaBAT2, 2) CONCOCT and MetaBAT2. Assessments were evaluated using AMBER (version 2.0.2). [file 40168_2022_1237_MOESM6_ESM.pdf]

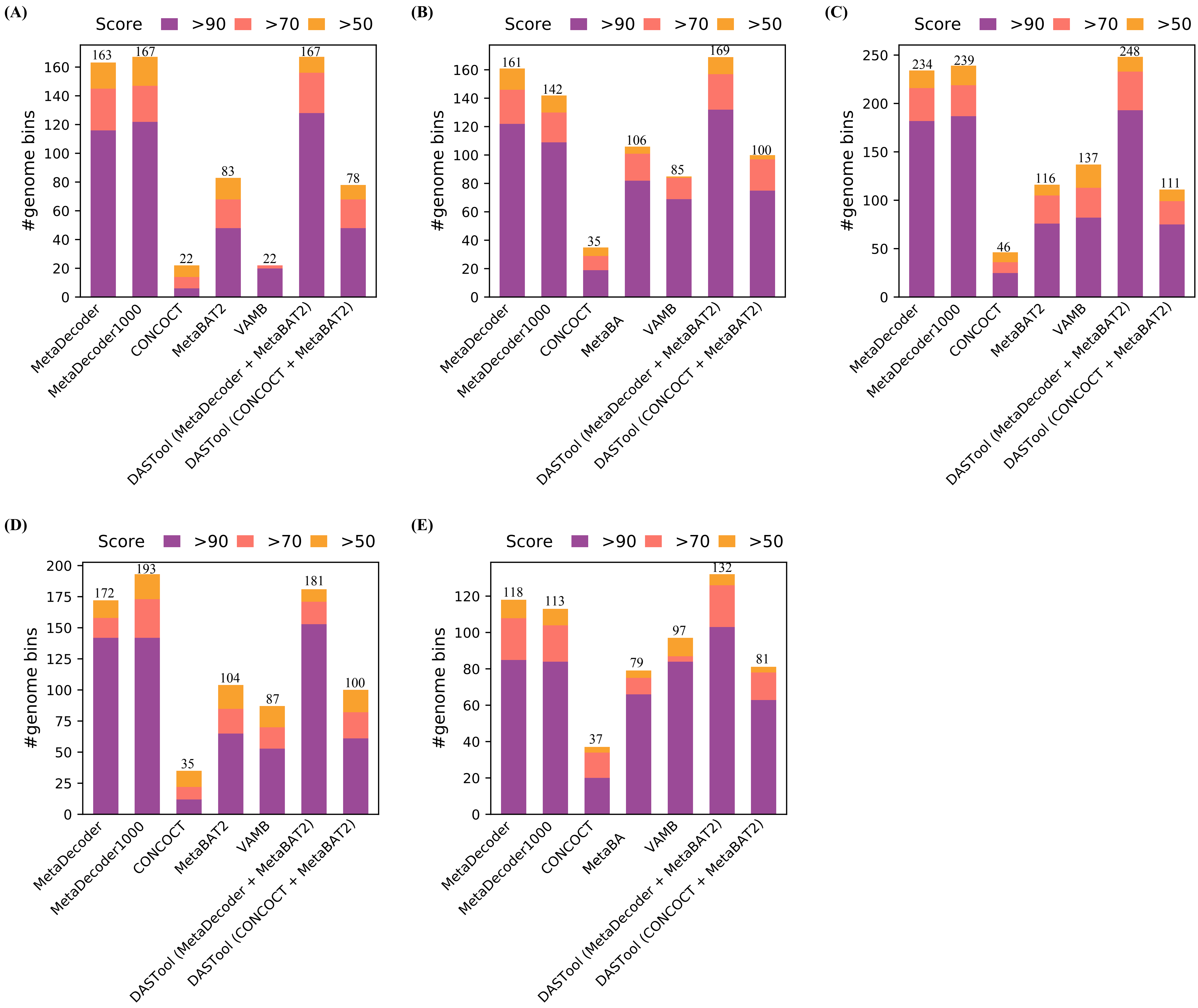

Supplementary Figure S6. Clustering benchmarks on five CAMI II Human Microbiome Project datasets. The number of identified bins on (*A*) Airways, (*B*) Gastrointestinal tract, (*C*) Oral cavity, (*D*) Skin and (*E*) Urogenital tract dataset with different score levels were shown. All programs were run in multi-sample mode with their default parameters. MetaDecoder with minimum sequence length setting to 1 Kb (MetaDecoder1000) was also added for benchmarking. DASTool was carried out on two combinations: 1) MetaDecoder and MetaBAT2, 2) CONCOCT and MetaBAT2. Assessments were evaluated using AMBER (version 2.0.2).
