# Supplementary material for: MetaDecoder: a novel method for clustering metagenomic contigs
Source: Microbiome. 2022 Mar 10;10:46. doi: 10.1186/s40168-022-01237-8 (PMC8908641; doi:10.1186/s40168-022-01237-8)
Supplement: Supplementary file 8 — Additional file 7: Supplementary Figure S7. Clustering benchmarks on 24 Human Microbiome Project datasets. The number of identified clusters with contamination ≤ 0.10 and different recall levels were shown. All programs were run with their default parameters. MetaDecoder with minimum sequence length setting to 1 Kb (MetaDecoder1000) was also added for benchmarking. Assessments were evaluated using CheckM (version 1.0.13). [file 40168_2022_1237_MOESM7_ESM.pdf]

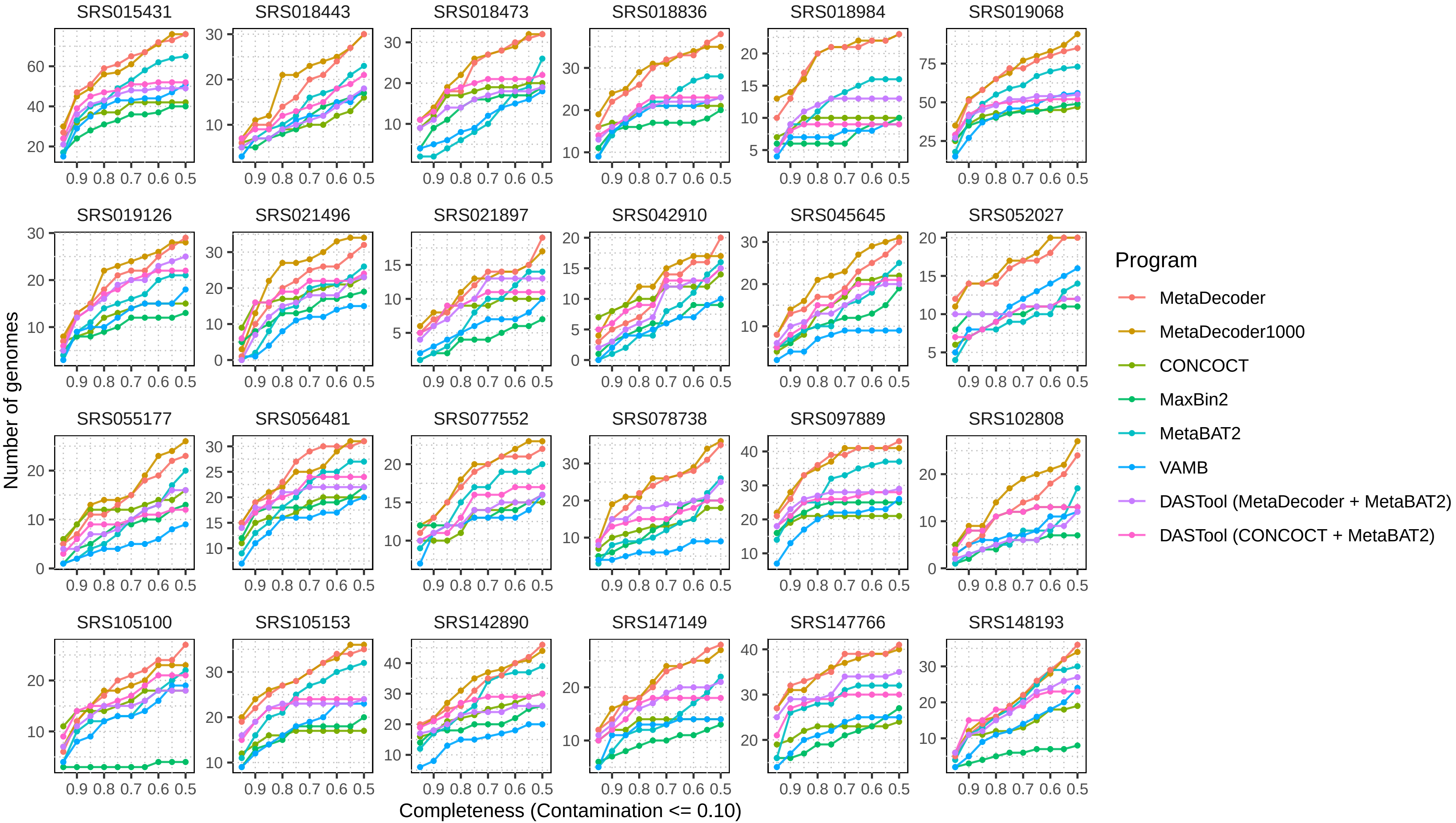

Supplementary Figure S7. Clustering benchmarks on 24 Human Microbiome Project datasets. The number of identified clusters with contamination ≤ 0.10 and different recall levels were shown. All programs were run with their default parameters. MetaDecoder with minimum sequence length setting to 1 Kb (MetaDecoder1000) was also added for benchmarking. Assessments were evaluated using CheckM (version 1.0.13).
