# Supplementary material for: MetaDecoder: a novel method for clustering metagenomic contigs
Source: Microbiome. 2022 Mar 10;10:46. doi: 10.1186/s40168-022-01237-8 (PMC8908641; doi:10.1186/s40168-022-01237-8)
Supplement: Supplementary file 11 — Additional file 10: Supplementary Figure S10. Phylogenetic analysis of 3,651 clusters with contamination ≤ 0.10 and completeness ≥ 0.50. Taxonomic labels were assigned by GTDB (version 95) using GTDB-TK (version 1.3.0) with default parameters. FastTree (version 2.1.11) with default parameters was used to construct the tree based on the concatenated marker gene amino acid alignments created by CheckM (version 1.0.13). The tree was visualized in iTOL. Only the bacterial phyla were shown. [file 40168_2022_1237_MOESM10_ESM.pdf]

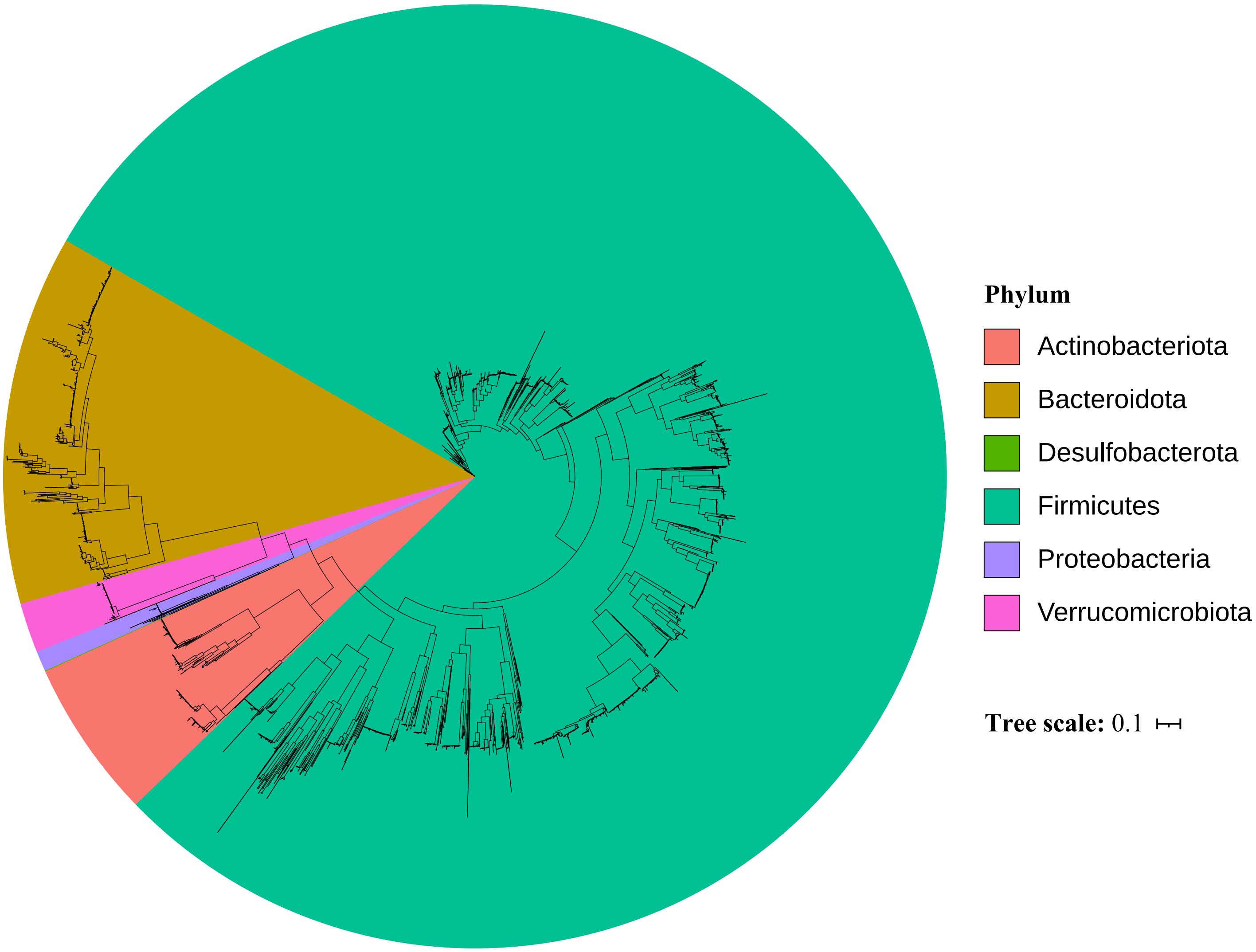

Supplementary Figure S10. Phylogenetic analysis of 3,651 clusters with contamination ≤ 0.10 and completeness ≥ 0.50. Taxonomic labels were assigned by GTDB (version 95) using GTDB-TK (version 1.3.0) with default parameters. FastTree (version 2.1.11) with default parameters was used to construct the tree based on the concatenated marker gene amino acid alignments created by CheckM (version 1.0.13). The tree was visualized in iTOL. Only the bacterial phyla were shown.
