# Supplementary material for: MetaDecoder: a novel method for clustering metagenomic contigs
Source: Microbiome. 2022 Mar 10;10:46. doi: 10.1186/s40168-022-01237-8 (PMC8908641; doi:10.1186/s40168-022-01237-8)
Supplement: Supplementary file 12 — Additional file 11: Supplementary Figure S11. Clustering benchmarks of MetaDecoder under different contig length thresholds. MetaDecoder with default length threshold (2.5 Kb) and 1 Kb (MetaDecoder1000) were applied on all real-world samples (i.e., two samples from high-CO2 cold-water geyser, 24 HMP samples and 145 samples from a cohort of T2D study), respectively. Cluster number (completeness ≥ 0.90) of each sample was shown at two contamination levels. Significance was determined using the Wilcoxon matched-pairs signed rank test. [file 40168_2022_1237_MOESM11_ESM.pdf]

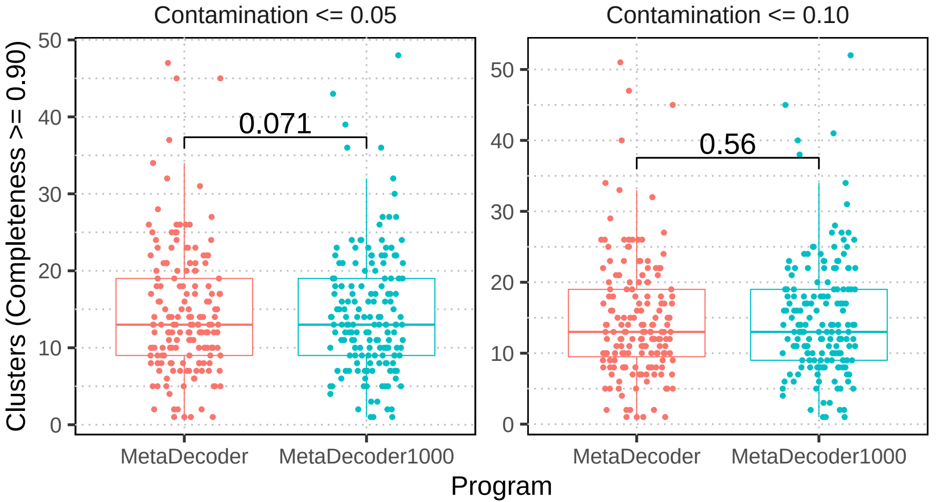

Supplementary Figure S11. Clustering benchmarks of MetaDecoder under different contig length thresholds. MetaDecoder with default length threshold (2.5 Kb) and 1 Kb (MetaDecoder1000) were applied on all real-world samples (*i.e.*, two samples from high-CO2 cold-water geyser, 24 HMP samples and 145 samples from a cohort of T2D study), respectively. Cluster number (completeness ≥ 0.90) of each sample was shown at two contamination levels. Significance was determined using the Wilcoxon matched-pairs signed rank test.
