# Supplementary material for: MetaDecoder: a novel method for clustering metagenomic contigs
Source: Microbiome. 2022 Mar 10;10:46. doi: 10.1186/s40168-022-01237-8 (PMC8908641; doi:10.1186/s40168-022-01237-8)
Supplement: Supplementary file 13 — Additional file 12: Supplementary Figure S12. Euclidean distance distributions between contigs abstracted from the same genome (intra-genome) and from two different genomes (inter-genome). The 3,023 bacterial genomes downloaded from NCBI were randomly fragmented into contigs (1 Kb - 100 Kb), then one million simulations were performed. Two histograms denote the intra- and interdistances, respectively. Two curves represent Gaussian densities parametrized by the mean and variance of the intra- and inter- distances, respectively. [file 40168_2022_1237_MOESM12_ESM.pdf]

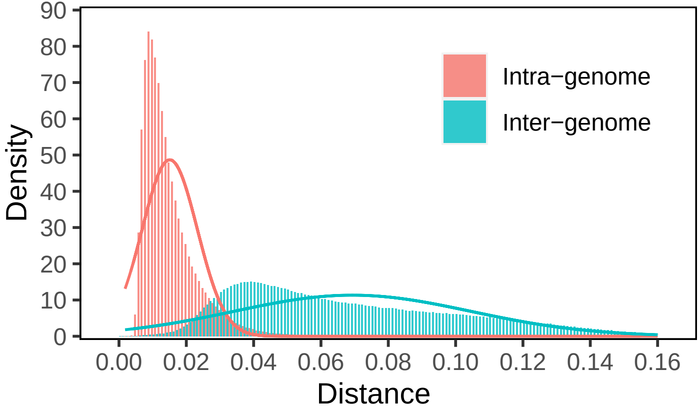

Supplementary Figure S12. Euclidean distance distributions between contigs abstracted from the same genome (intra-genome) and from two different genomes (inter-genome). The 3,023 bacterial genomes downloaded from NCBI were randomly fragmented into contigs (1 Kb - 100 Kb), then one million simulations were performed. Two histograms denote the intra- and inter-distances, respectively. Two curves represent Gaussian densities parametrized by the mean and variance of the intra- and inter- distances, respectively.
